# Age and sex are associated with the plasma lipidome: findings from the GOLDN study

**DOI:** 10.1186/s12944-021-01456-2

**Published:** 2021-04-03

**Authors:** Emily Slade, Marguerite R. Irvin, Kevin Xie, Donna K. Arnett, Steven A. Claas, Tobias Kind, David W. Fardo, Gregory A. Graf

**Affiliations:** 1 Department of Biostatistics, University of Kentucky, 725 Rose St, Multidisciplinary Science Building, Suite 205, Lexington, KY 40536 USA; 2 Department of Epidemiology, University of Alabama at Birmingham, Birmingham, AL USA; 3 College of Public Health, University of Kentucky, Lexington, KY USA; 4 West Coast Metabolomics Center, University of California, Davis, CA USA; 5 Department of Pharmaceutical Sciences, University of Kentucky, Lexington, KY USA

**Keywords:** Lipidomics, Glycerolipids, Glycerophospholipids, Sphingomyelin, Sterols, Fatty acid, Acylcarnitines, Cohort, Age, Sex

## Abstract

**Background:**

Developing an understanding of the biochemistry of aging in both sexes is critical for managing disease throughout the lifespan. Lipidomic associations with age and sex have been reported, but prior studies are limited by measurements in serum rather than plasma or by participants taking lipid-lowering medications.

**Methods:**

Our study included lipidomic data from 980 participants aged 18–87 years old from the Genetics of Lipid-Lowering Drugs and Diet Network (GOLDN). Participants were off lipid-lowering medications for at least 4 weeks, and signal intensities of 413 known lipid species were measured in plasma. We examined linear age and sex associations with signal intensity of (a) 413 lipid species; (b) 6 lipid classes (glycerolipids, glycerophospholipids, sphingolipids, sterol lipids, fatty acids, and acylcarnitines); and (c) 15 lipid subclasses; as well as with the particle sizes of three lipoproteins.

**Results:**

Significant age associations were identified in 4 classes, 11 subclasses, 147 species, and particle size of one lipoprotein while significant sex differences were identified in 5 classes, 12 subclasses, 248 species, and particle sizes of two lipoproteins. For many lipid species (*n* = 97), age-related associations were significantly different between males and females. Age*sex interaction effects were most prevalent among phosphatidylcholines, sphingomyelins, and triglycerides.

**Conclusion:**

We identified several lipid species, subclasses, and classes that differ by age and sex; these lipid phenotypes may serve as useful biomarkers for lipid changes and associated cardiovascular risk with aging in the future. Future studies of age-related changes throughout the adult lifespan of both sexes are warranted.

**Trial registration:**

ClinicalTrials.gov NCT00083369; May 21, 2004.

**Supplementary Information:**

The online version contains supplementary material available at 10.1186/s12944-021-01456-2.

## Introduction

Global life expectancy has increased [1] and is predicted to continue increasing in many countries [2]. In developed nations, age is the primary risk factor for many common diseases including cardiovascular disease, cancer, and Alzheimer’s disease [3]. Human aging is a complex phenomenon influenced by environmental, genetic and other endogenous factors. For example, the aging process differs between sexes, and there are well established associations between sex and the incidence, symptoms, severity, age of onset, and response to treatments for many diseases. Management of disease in aging populations may be improved by better understanding the physiology of aging in both sexes. Given the complex biochemistry of aging, metabolomic approaches may offer important insights into the aging process and age-related disease etiologies. Lipidomics, in particular, is well positioned to investigate those pathologies. Lipids function as both structural and signaling molecules and are known to play important roles in age-related disorders such as cardiovascular disease [4], metabolic syndrome [5], macular degeneration [6], neurodegenerative diseases [7], and at least some types of stroke [8]. However, before lipidomics can be used to dissect and characterize disease, it is critical that we understand how the lipidome changes during normal, healthy aging in both sexes.

A number of well-powered metabolomic studies of age and sex have included lipidomic species [9–12]; however, most of these studies measured metabolites in serum, while current recommendations favor plasma for lipidomics due to the generation and degeneration of lipid species during the coagulation process and the similarity of plasma generated from fresh blood to the plasma compartment in vivo [13]. Beyene et al. studied a large Australian cohort (*n* = 10,339) to examine age and sex associations with individual plasma lipid species that are independent of lipoprotein metabolism [12]. Wong et al. conducted a plasma lipidomics study in a modestly sized sample (*n* = 100) of participants age > 55 years [14]. Darst et al. conducted a longitudinal plasma lipidomics analysis of 1212 participants with up to three study visits per participant, with follow-up occurring four years after baseline and every two years thereafter [15, 16]. However, these studies were not restricted to participants free of lipid-lowering medication, somewhat complicating the interpretation of their findings.

The Genetics of Lipid-Lowering Drugs and Diet Network (GOLDN) offers a context to investigate lipidomic markers of age and sex. Data on GOLDN’s 980 participants includes robust characterization of health status, diet, anthropometry, and demographics. No GOLDN participants were taking lipid-lowering drugs, including fish oils, at the time specimens were collected, thus avoiding the difficulties of interpretation that have characterized some previous studies. GOLDN participants ranged in age from 18 to 87 years, making it better suited for a study across the adult lifespan than previous studies with a more age-restricted sample. In this study we used liquid chromatography/mass spectrometry to quantify 413 lipidomic species in GOLDN plasma specimens. The goal of this study is to examine associations between age and each lipid species and to assess whether these associations across the adult lifespan differ between sexes.

## Methods

The GOLDN Study has been described in detail elsewhere [17]. GOLDN participants were re-recruited from three-generational pedigrees from two National Heart, Lung and Blood Institute Family Heart Study field centers (Minneapolis, MN and Salt Lake City, UT); genetically related pedigree members of these probands were recruited as well. Nearly all participants were of European ancestry. Earlier studies demonstrated that Caucasians in Utah and Minnesota were homogeneous, and pooling data across centers did not threaten the validity of this study [18].

Exclusion criteria included the following: recent history (past six months) of myocardial infarction, coronary bypass surgery, coronary angioplasty or percutaneous transluminal coronary angioplasty; current use of warfarin or insulin; self-report of a positive history of kidney, gall bladder, liver, or pancreatic disease, or a history of nutrient malabsorption; serum concentrations of alanine transaminase exceeding 66 U/L in males or 44 U/L in females; serum concentrations of aspartate aminotransferase exceeding 52 U/L in males or 42 U/L in females; glomerular filtration rate < 30 ml/min/1.73 m^2^ estimated from the Modification of Diet in Renal Disease (MDRD) Study equation; fasting triglycerides ≥1500 mg/dL; pregnant women or women of childbearing potential not using contraception; and women nursing a child. Individuals who reported current use of prescription and/or over-the-counter hypolipidemic drugs or dietary supplements known to influence lipid values (e.g., fish oil, niacin, flaxseed oil) were required to consult with their clinician for approval to discontinue these lipid-lowering agents for four weeks prior to study participation.

Specimens were collected at a pre-intervention, baseline visit. Participants were asked to fast for ≥12 h and abstain from using alcohol for ≥24 h before the clinic visit [19]. Blood samples were centrifuged within 20 min of collection at 2000 g for 15 min at 4 °C [20]. Plasma samples were stored at − 80 °C.

Unique, known lipid species were analyzed by liquid chromatography coupled-mass spectrometry (LC-MS/MS). The data set was obtained as an untargeted lipidomic profiling experiment. Differences in signal intensity reflect differences in abundance of any given lipid species based on instrumental settings and solvents and buffers used during analysis. The relative concentrations are comparable within the experiments obtained with the same protocol but, by design, cannot be easily compared with absolute (targeted) measurements. The signals of lipids designated as (A) and (B) are related to double bond stereoisomers (cis/trans) which can be resolved by the chromatographic protocol used. Due to missing authentic reference compounds, no further annotations can be made. Moreover, tetrahedral stereoisomers (R/S) and regioisomers were not distinguished. Raw lipidomic data were normalized using Systematic Error Removal using Random Forest (SERRF) [21]. SERRF is a machine learning algorithm for large metabolomic data sets that characterizes systematic error such as batch effects and day-to-day variation in quality control samples and uses this model to reduce systematic error in study samples [21]. For lipid species measured on both positive and negative modes, the mode with the smaller standard deviation across participants was retained for analysis.

For statistical analysis, individual lipid species were classified into six major classes: glycerolipids, glycerophospholipids, sphingolipids, sterol lipids, fatty acids, and acylcarnitines. Four of the six major classes were further subdivided into 15 subclasses: glycerolipids were subdivided into triglycerides (TG) and diacylglycerols (DG); glycerophospholipids were subdivided into phosphatidylcholines (PC), phospatidylethanolamines (PE), phosphatidylinositols (PI), phosphatidylglycerols (PG), lysophosphatidylethanolamines (LPE), and lysophosphatidylcholines (LPC); sphingolipids were subdivided into sphingomyelins (SM), ceramides, lactosylceramides (LCer), glucosylceramides (GlcCer), and galactosylgalactosylceramides (GalGalCer); sterol lipids were subdivided into cholesterol and cholesteryl esters (CE). Fatty acids and acylcarnitines were not further subdivided. Analyses were performed at the class level, subclass level, and individual lipid level. For class and subclass analyses, each participant’s observed class/subclass intensity was calculated by summing the total signal intensity of lipid species belonging to the class/subclass for that participant.

Three sets of analyses were performed to examine the associations of age, sex, and their interaction with signal intensities of (1) classes of lipids, (2) subclasses of lipids, and (3) individual lipid species. The class intensities, subclass intensities, and individual lipid intensities were standardized to have mean 0 and standard deviation 1 prior to analysis. This standardization of outcome data allows for a direct comparison of estimated regression coefficients between models. Further, it aids in interpretation as a one unit change in outcome reflects a change of one standard deviation in signal intensity for all lipid species, classes, and subclasses. No further transformation was performed to the classes, subclasses, and individual lipid species.

A fourth set of analyses was also performed to examine the associations of age, sex, and their interaction with lipoprotein particle size measured by nuclear magnetic resonance (NMR) spectroscopy. Lipoproteins measured via NMR spectroscopy include very-low-density lipoprotein (VLDL), low-density lipoprotein (LDL), and high-density lipoprotein (HDL). All particle sizes are measured in nanometers (nm).

All analyses employed linear regression modeling to examine the associations between the class, subclass, lipid species, or lipoprotein particle size outcome (each separately) with age, sex, and age*sex interaction, while including adjustment for body mass index (BMI). Other studies have shown BMI associations with the plasma lipidome [22, 23]. As such, BMI is included as a covariate in all analyses in order to examine age and sex associations with the plasma lipidome that are not driven by BMI-related associations. Analyses of classes, subclasses, and individual lipid species also included adjustment for batch effects. For analyses including an age*sex interaction, the age variable was centered prior to analysis to aid in interpretability of the interaction term. For classes, subclasses, individual lipid species, and lipoprotein particle sizes exhibiting significant age*sex interaction effects, age associations with the class, subclass, lipid species, or lipoprotein particle size outcome were further explored in sex-stratified analyses, i.e., separate linear regression models were fit for males and females to explore the relationship between age and the class, subclass, lipid species, or lipoprotein particle size in a more easily interpretable form.

To account for multiple testing, a Benjamini-Hochberg adjustment (false discovery rate correction) was applied to control the false discovery rate in each set of analyses at 0.05 [24]. All reported *P*-values are the Benjamini-Hochberg-adjusted *P*-values, and thus, a significance level of 0.05 was utilized for all analyses. All analyses were performed using R version 4.0.2 [25].

## Results

### Participant demographics

Fasting plasma samples were analyzed for 980 participants with 413 unique, known lipid species identified. The mean age of participants was 48.3 years (standard deviation = 16.4 years), and 52.3% were female (Table 1). As expected by design of the GOLDN study, participants identified primarily as white (99.9%) (Table 1).

**Table 1:** GOLDN cohort characteristics, mean (standard deviation) or n (%)

	All Participants, *n* = 980	Male, *n* = 467	Female, *n* = 513
Age (years)	48.3 (16.4)	48.8 (16.4)	48.0 (16.4)
White race	979 (99.9%)	466 (99.8%)	513 (100.0%)
Alcohol consumer	477 (48.7%)	220 (47.1%)	257 (50.1%)
Smoking status			
Current smoker	73 (7.4%)	36 (7.7%)	37 (7.2%)
Past smoker	211 (21.5%)	121 (25.9%)	90 (17.5%)
Never smoker	695 (70.9%)	310 (66.4%)	385 (75.0%)
Missing	1 (0.1%)	0 (0.0%)	1 (0.2%)
BMI (kg/m^2^)	28.3 (5.7)	28.4 (4.8)	28.1 (6.3)
Waist-hip ratio	0.9 (0.1)	0.9 (0.1)	0.9 (0.1)
Blood glucose (mg/dL)	97.5 (15.3)	100.8 (16.2)	94.4 (13.8)
LDL cholesterol (mg/dL)	121.8 (30.9)	123.4 (30.1)	120.5 (31.6)
HDL cholesterol (mg/dL)	47.1 (13.1)	41.3 (9.6)	52.5 (13.6)
Triglycerides (mg/dL)	138.6 (96.8)	151.3 (111.0)	127.1 (80.3)
Creatine (mg/dL)	0.8 (0.2)	0.9 (0.2)	0.7 (0.1)
HOMA-IR (score)	3.5 (2.4)	3.7 (2.6)	3.3 (2.3)
Coronary heart disease	49 (5.0%)	41 (8.8%)	8 (1.6%)
Diabetes	75 (7.7%)	31 (6.6%)	44 (8.6%)
Hypertension	256 (26.1%)	127 (27.2%)	129 (25.1%)

Summaries of the characteristics of the GOLDN cohort, both overall and stratified by sex, are provided as mean (standard deviation) for numerical variables or as n (%) for categorical variables

### Age and sex are associated with lipid classes

Among the six classes of lipid species, four had significant associations between age and total class intensity after adjustment for sex, BMI, and batch (Table 2). These include glycerophospholipids, sphingolipids, sterol lipids, and fatty acids, which all increase significantly with age. On average, total intensity of glycerolipids and acylcarnitines did not change significantly with age after adjustment for sex, BMI, and batch (Table 2). Five of the six classes of lipid species had a significant association with sex (Table 2). Total intensities of glycerophospholipids, sphingolipids, sterol lipids, and fatty acids were significantly higher in women than men, and total intensity of glycerolipids was significantly higher in men than women after adjustment for age, BMI, and batch. The average total intensity of acylcarnitines did not differ significantly between males and females after adjustment for age, BMI, and batch (Table 2).

**Table 2:** Associations between total class intensity with age, sex, and their interaction

Class	Age β (SE)	Sex β (SE)	Age*Sex Interaction β (SE)
Glycerolipids	0.0050 (0.0027)	− 0.2036 (0.0600)^b^	0.0098 (0.0037)^a^
Glycerophospholipids	0.0132 (0.0026)^c^	0.3243 (0.0580)^c^	0.0116 (0.0035)^b^
Sphingolipids	0.0178 (0.0026)^c^	0.1657 (0.0577)^b^	0.0074 (0.0035)
Sterol lipids	0.0094 (0.0028)^b^	0.2467 (0.0624)^c^	0.0002 (0.0038)
Fatty acids	0.0067 (0.0027)^a^	0.2898 (0.0597)^c^	−0.0017 (0.0036)
Acylcarnitines	0.0042 (0.0028)	0.0485 (0.0629)	−0.0012 (0.0038)

Rows include regression coefficients (β) and standard errors from separate linear regression models with outcome of standardized total class intensity. Each model includes age, sex, age*sex interaction, batch, and BMI as covariates. In all models, age is centered at the mean age of 48.3 years, and the reference category for sex is male. All *P*-values are adjusted for multiple testing using a Benjamini-Hochberg adjustment to control the false discovery rate among each set of six coefficients at 0.05. Age coefficients (β) can be interpreted as the expected change in standardized total class intensity for a one-year increase in age, among men, after adjustment for batch and BMI. Sex coefficients (β) can be interpreted as the expected difference in standardized total class intensity between women and men, at the mean age, after adjustment for batch and BMI (positive values indicate higher expected levels in women). Age*sex interaction coefficients (β) can be interpreted as the expected additional change in standardized total class intensity for a one-year increase in age, among women (on top of the age coefficient for men), after adjustment for batch and BMI. Put more simply, add the age coefficient and age*sex interaction coefficient together to find the expected change in standardized total class intensity for a one-year increase in age, among women, after adjustment for batch and BMI

^a^adjusted *P*-value < 0.05

^b^adjusted *P*-value < 0.01

^c^adjusted *P*-value < 0.001

**Fig. 1:**
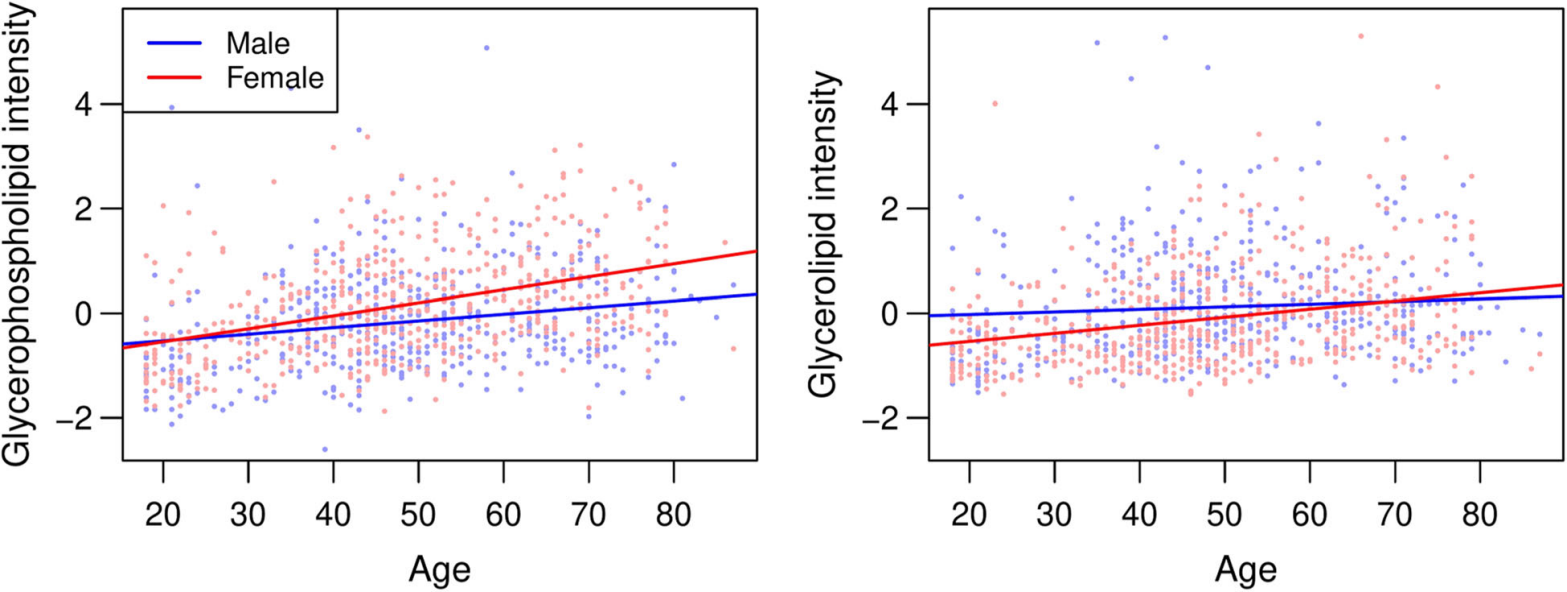
Relationship between age and standardized total class intensity for glycerophospholipids (left) and glycerolipids (right). Dots represent observed values for males (blue) and females (red). Lines represent a linear regression of class intensity on age, performed separately for males (blue) and females (red). Only lipid classes with significant age*sex interaction effects from the overall analyses (see Table 2) are analyzed in these stratified models

In two classes of lipids, glycerophospholipids (*P* = 0.007) and glycerolipids (*P* = 0.022), age and sex had a significant interaction effect, indicating that the association between age and the total class intensity was different among males and females, while controlling for BMI and batch (Table 2). In both classes, the expected increase in the total class intensity with increasing age was larger in women than in men (Fig. 1). For a ten-year increase in age, our model predicts the total intensity of glycerophospholipids to increase by 0.05 standard deviations in men and by 0.15 standard deviations in women after controlling for BMI and batch (Table 2, Fig. 1). For a ten-year increase in age, we expect the total intensity of glycerolipids to increase by 0.13 standard deviations in men and by 0.25 standard deviations in women after controlling for BMI and batch (Table 2, Fig. 1). Correlations between total intensities of the six lipid classes are shown in Additional File 2.

### Age and sex are associated with lipid subclasses

Among the 15 subclasses of lipid species, 11 had significant associations with age after adjustment for sex, BMI, and batch (Table 3). Of these, cholesterol was the only subclass that decreased significantly with age. The other 10 (PC, PE, PI, PG, LPE, SM, ceramide, LCer, GlcCer, CE) all increased with age. Of the 15 subclasses of lipid species, 12 had significant associations with sex after adjustment for age, BMI, and batch (Table 3). On average, women had higher total subclass intensity of PC, PE, PI, PG, SM, GlcCer, and CE, while men had higher total subclass intensity of TG, DG, LPC, ceramides, and cholesterol after adjustment for age, BMI, and batch (Table 3).

**Table 3:** Associations between total subclass intensity with age, sex, and their interaction

Subclass	Age β (SE)	Sex β (SE)	Age*Sex Interaction β (SE)
TG	0.0050 (0.0027)	−0.2035 (0.0600)^b^	0.0098 (0.0037)^a^
DG	0.0055 (0.0027)	−0.1708 (0.0612)^b^	0.0082 (0.0037)
PC	0.0148 (0.0026)^c^	0.3695 (0.0574)^c^	0.0106 (0.0035)^a^
PE	0.0057 (0.0026)^a^	0.3079 (0.0584)^c^	0.0116 (0.0036)^b^
PI	0.0072 (0.0027)^a^	0.2908 (0.0606)^c^	0.0070 (0.0037)
PG	0.0103 (0.0026)^c^	0.2559 (0.0569)^c^	0.0023 (0.0035)
LPE	0.0059 (0.0028)^a^	−0.1244 (0.0618)	0.0072 (0.0038)
LPC	0.0006 (0.0027)	−0.5470 (0.0606)^c^	0.0094 (0.0037)^a^
SM	0.0152 (0.0026)^c^	0.3631 (0.0587)^c^	0.0082 (0.0036)
Ceramide	0.0175 (0.0026)^c^	−0.1335 (0.0579)^a^	0.0046 (0.0035)
LCer	0.0109 (0.0028)^c^	0.0242 (0.0624)	−0.0008 (0.0038)
GlcCer	0.0108 (0.0022)^c^	0.1240 (0.0499)^a^	0.0061 (0.0030)
GalGalCer	0.0000 (0.0027)	−0.0223 (0.0602)	−0.0130 (0.0037)^b^
Cholesterol	−0.0123 (0.0027)^c^	−0.2907 (0.0595)^c^	0.0012 (0.0036)
CE	0.0101 (0.0028)^c^	0.2637 (0.0623)^c^	0.0002 (0.0038)

Rows include regression coefficients (β) and standard errors from separate linear regression models with outcome of standardized total subclass intensity. Each model includes age, sex, age*sex interaction, batch, and BMI as covariates. In all models, age is centered at the mean age of 48.3 years, and the reference category for sex is male. All *P*-values are adjusted for multiple testing using a Benjamini-Hochberg adjustment to control the false discovery rate among each set of 15 coefficients at 0.05. Age coefficients (β) can be interpreted as the expected change in standardized total subclass intensity for a one-year increase in age, among men, after adjustment for batch and BMI. Sex coefficients (β) can be interpreted as the expected difference in standardized total subclass intensity between women and men, at the mean age, after adjustment for batch and BMI (positive values indicate higher expected levels in women). Age*sex interaction coefficients (β) can be interpreted as the expected additional change in standardized total subclass intensity for a one-year increase in age, among women (on top of the age coefficient for men), after adjustment for batch and BMI. Put more simply, add the age coefficient and age*sex interaction coefficient together to find the expected change in standardized total subclass intensity for a one-year increase in age, among women, after adjustment for batch and BMI

^a^adjusted *P*-value < 0.05

^b^adjusted *P*-value < 0.01

^c^adjusted *P*-value < 0.001

**Fig. 2:**
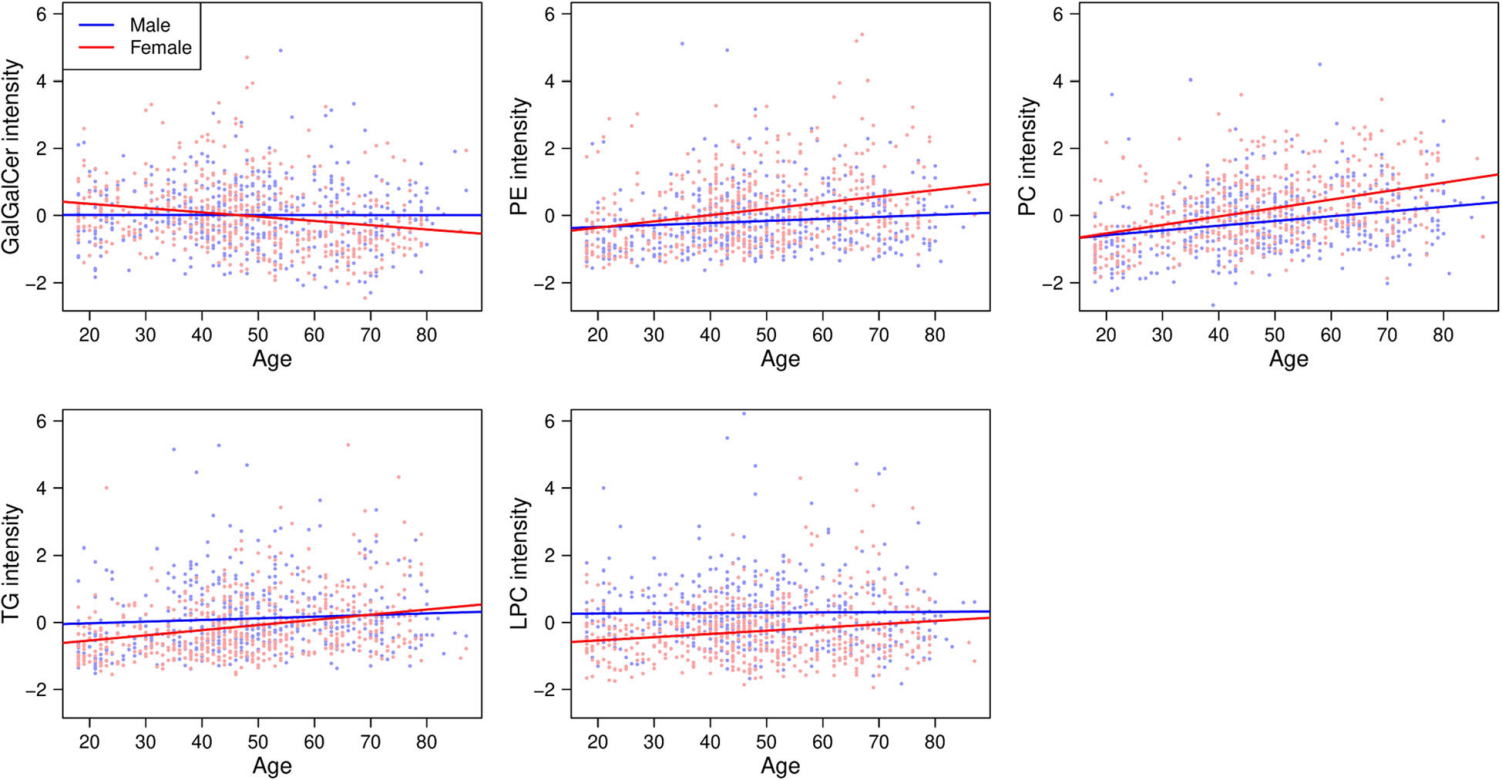
Relationship between age and standardized total subclass intensity. Dots represent observed values for males (blue) and females (red). Lines represent a linear regression of subclass intensity on age, performed separately for males (blue) and females (red). Only lipid subclasses with significant age*sex interaction effects from the overall analyses (see Table 3) are analyzed in these stratified models. These include galactosylgalactosylceramides (top left), phosphatidylethanolamines (top center), phosphatidylcholines (top right), triglycerides (bottom left), and lysophosphatidylcholines (bottom center)

Five subclasses of lipid species exhibited a significant interaction effect between age and sex (Table 3). These included GalGalCer (*P* = 0.006), PE (*P* = 0.008), PC (*P* = 0.012), TG (*P* = 0.028), and LPC (*P* = 0.033). In GalGalCer and LPC, the total intensity remained approximately constant across ages in men, but in women there was a significant decrease in GalGalCer with age and a significant increase in LPC with age (Table 3, Fig. 2). In the PE, PC, and TG subclasses, total subclass intensity increased with age in both men and women, but the rate of increase was larger in women than men after controlling for BMI and batch (Table 3, Fig. 2).

### Age and sex are associated with individual lipid species

Among the 413 individual lipid species, 147 had significant associations with age after adjustment for sex, BMI, and batch (Table 4, Additional File 1); on average, 141 increased with age, and 6 decreased with age. Among the 413 individual lipid species, 248 had a significant association with sex after adjustment for age, BMI, and batch (Table 4, Additional File 1). Of these, 103 lipids had higher levels in males and 145 had higher levels in females, on average (Table 4, Additional File 1).

**Table 4:** Associations between lipid signal intensity with age, sex, and their interaction (includes those with top 10 most significant age*sex interactions)

Lipid Species	Age β (SE)	Sex β (SE)	Age*Sex Interaction β (SE)
PC (p-38:5) or PC (o-38:6) A	0.0071 (0.0026)^a^	0.2561 (0.0587)^c^	0.0201 (0.0036)^c^
PC (38:4) A	0.0026 (0.0027)	0.2269 (0.0595)^c^	0.0192 (0.0036)^c^
PC (34:4)	−0.0022 (0.0027)	0.5713 (0.0598)^c^	0.0163 (0.0036)^c^
PC (38:4)	−0.0018 (0.0027)	0.1720 (0.0611)^b^	0.0169 (0.0037)^c^
SM (d32:2)	0.0048 (0.0022)	1.0661 (0.0485)^c^	0.0131 (0.0030)^c^
SM (d41:2) B	0.0063 (0.0026)^a^	0.6994 (0.0581)^c^	0.0157 (0.0035)^c^
PC (35:4)	0.0006 (0.0027)	0.4934 (0.0608)^c^	0.0156 (0.0037)^b^
SM (d30:1)	0.0029 (0.0026)	0.7290 (0.0580)^c^	0.0140 (0.0035)^b^
TG (56:5) B	0.0046 (0.0027)	−0.1895 (0.0595)^b^	0.0145 (0.0036)^b^
LPC (22:5)	−0.0049 (0.0027)	−0.5314 (0.0609)^c^	0.0146 (0.0037)^b^

Rows include regression coefficients (β) and standard errors from separate linear regression models with outcome of standardized lipid intensity. Only results from models exhibiting the top 10 most significant age*sex interactions are shown; a full table of results for all 413 models is included in Additional File 1. Each model includes age, sex, age*sex interaction, batch, and BMI as covariates. In all models, age is centered at the mean age of 48.3 years, and the reference category for sex is male. All *P*-values are adjusted for multiple testing using a Benjamini-Hochberg adjustment to control the false discovery rate among each set of 413 coefficients at 0.05. Age coefficients (β) can be interpreted as the expected change in standardized lipid intensity for a one-year increase in age, among men, after adjustment for batch and BMI. Sex coefficients (β) can be interpreted as the expected difference in standardized lipid intensity between women and men, at the mean age, after adjustment for batch and BMI (positive values indicate higher expected levels in women). Age*sex interaction coefficients (β) can be interpreted as the expected additional change in standardized lipid intensity for a one-year increase in age, among women (on top of the age coefficient for men), after adjustment for batch and BMI. Put more simply, add the age coefficient and age*sex interaction coefficient together to find the expected change in standardized lipid intensity for a one-year increase in age, among women, after adjustment for batch and BMI. Lipids (A) and (B) designate resolved cis/trans stereoisomers

^a^adjusted *P*-value < 0.05

^b^adjusted *P*-value < 0.01

^c^adjusted *P*-value < 0.001

**Fig. 3:**
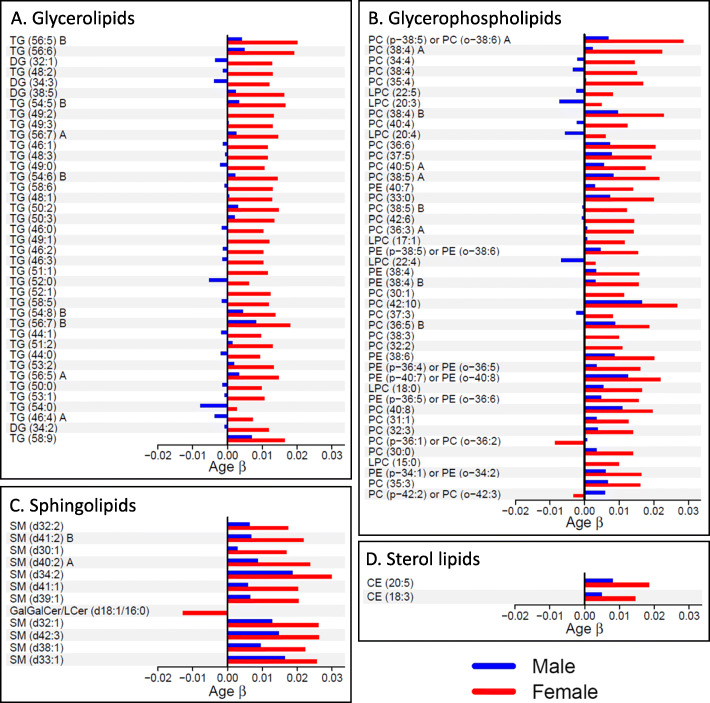
Relationship between age and standardized individual lipid intensity, stratified by sex. Bars indicate the age coefficient, representing the expected change in standardized lipid intensity for a one-year increase in age, from separate linear regression models for males (blue) and females (red). Only lipid species with significant age*sex interaction effects from the overall analyses (see Table 4) are analyzed in these stratified models. Lipid species are grouped by class for ease of presentation (panel **A**: glycerolipids, panel **B**: glycerophospholipids, panel **C**: sphingolipids, panel **D**: sterol lipids); within each class, lipids are ordered by size of the age*sex interaction effect (see Table 4). Lipids denoted A and B designate resolved cis/trans stereoisomers

For 97 individual lipid species, the association between age and lipid intensity was significantly different between males and females after adjustment for batch and BMI (Table 4, Additional File 1). All but three of these significant age*sex interaction effects were in the form of larger age-related increases in lipid intensity in females as compared to males (Fig. 3). Only PC (p-36:1) or PC (o-36:2), GalGalCer/LCer (d18:1/16:0), and PC (p-42:2) or PC (o-42:3) deviated from this pattern; the first two exhibited age-related declines in females with very little change in males, and the last exhibited an age-related decline in females and an age-related increase in males.

### Analysis of lipoprotein particle sizes

**Fig. 4:**
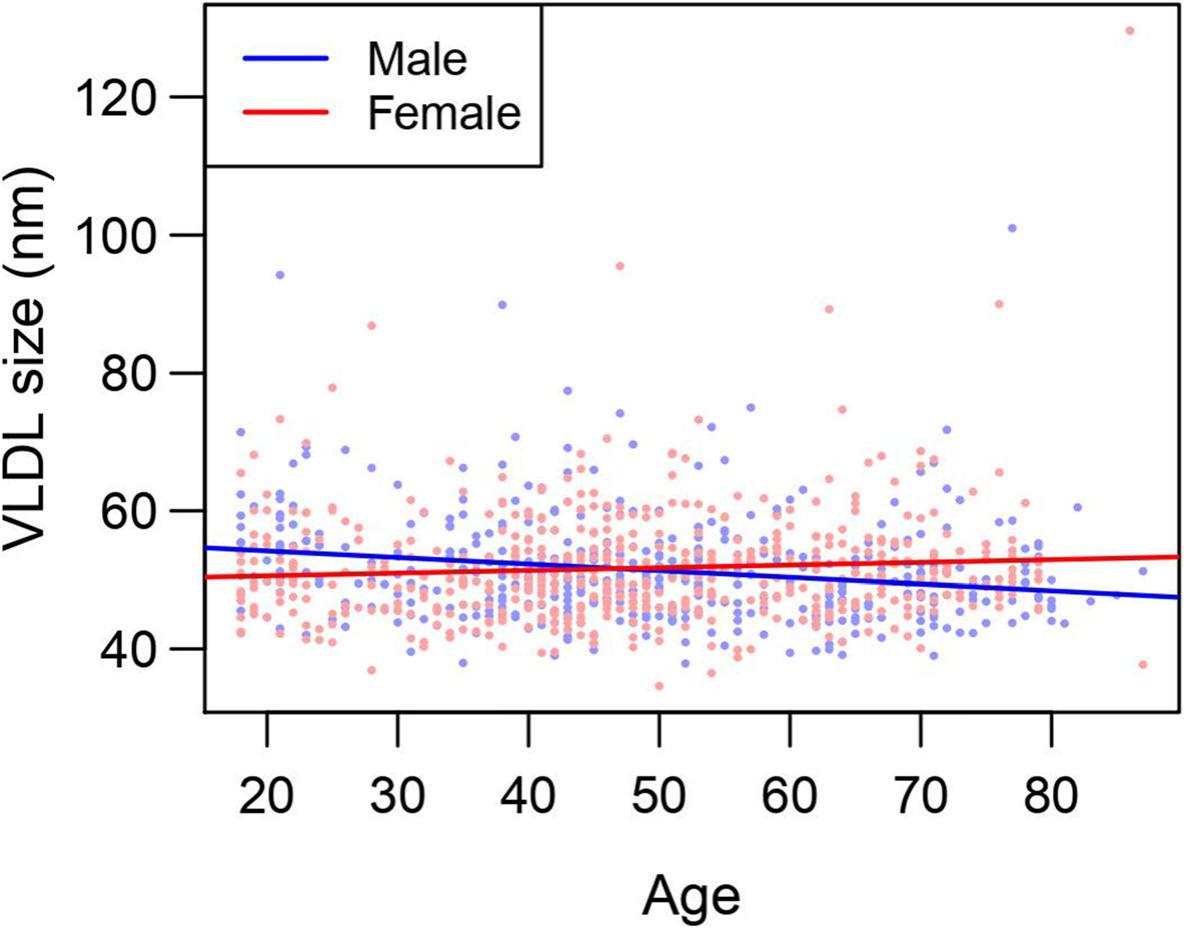
Relationship between age and lipoprotein particle size, stratified by sex. Dots represent observed values for males (blue) and females (red). Lines represent a linear regression of lipoprotein particle size on age, performed separately for males (blue) and females (red). Only particles with significant age*sex interaction effects from the overall analyses (see Table 5) are analyzed in these stratified models. This includes only VLDL

Of the three lipoproteins measured via NMR spectroscopy, only VLDL exhibited a significant association with age after adjustment for sex and BMI (Table 5). This association between age and VLDL particle size was also significantly different between males and females after adjustment for BMI (Table 5, Fig. 4). In males, VLDL particle size decreased with age by an average of 0.090 nm per year; and in females, VLDL particle size slightly increased with age by an average of 0.036 nm per year, after adjusting for BMI (Table 5).

**Table 5:** Associations between lipoprotein particle size with age, sex, and their interaction

Lipoprotein Particle	Age β (SE)	Sex β (SE)	Age*Sex Interaction β (SE)
VLDL size (nm)	−0.090 (0.023)^c^	0.256 (0.504)	0.126 (0.031)^c^
LDL size (nm)	−0.003 (0.002)	0.587 (0.049)^c^	0.003 (0.003)
HDL size (nm)	0.001 (0.001)	0.359 (0.025)^c^	0.001 (0.002)

Rows include regression coefficients (β) and standard errors from separate linear regression models with outcome of lipoprotein particle size (measured via nuclear magnetic resonance (NMR) spectroscopy). Each model includes age, sex, age*sex interaction, and BMI as covariates. In all models, age is centered at the mean age of 48.3 years, and the reference category for sex is male. All *P*-values are adjusted for multiple testing using a Benjamini-Hochberg adjustment to control the false discovery rate among each set of three coefficients at 0.05. Age coefficients (β) can be interpreted as the expected change in particle size (nm) for a one-year increase in age, among men, after adjustment for BMI. Sex coefficients (β) can be interpreted as the expected difference in particle size (nm) between women and men, at the mean age, after adjustment for BMI (positive values indicate larger expected size in women). Age*sex interaction coefficients (β) can be interpreted as the expected additional change in particle size (nm) for a one-year increase in age, among women (on top of the age coefficient for men), after adjustment for BMI. Put more simply, add the age coefficient and age*sex interaction coefficient together to find the expected change in particle size (nm) for a one-year increase in age, among women, after adjustment for BMI

^a^adjusted *P*-value < 0.05

^b^adjusted *P*-value < 0.01

^c^adjusted *P*-value < 0.001

There was not a significant association between LDL and HDL particle size with age, but both lipoprotein particle sizes were significantly different between males and females after adjustment for BMI (Table 5). On average, LDL particles were 0.587 nm larger in females than in males, and HDL particles were 0.359 nm larger in females than in males, after adjustment for BMI (Table 5).

## Discussion

Findings from the GOLDN study indicated many age and sex-related associations in the plasma lipidome in a large (*n* = 980) cohort of participants. GOLDN reflects a unique cohort for lipidomic analyses because all participants were treatment-naïve at the time of plasma collection and therefore, results are not confounded by the effects of lipid-lowering drugs. Many significant differences in the lipidomic measures were identified in the lipid classes, lipid subclasses, and individual lipid species for age and sex. These findings are highly consistent with the results from the Australian diabetes (AusDiab) cohort study which evaluated 706 lipids species in plasma; of the 11 subclasses reported in GOLDN to differ by sex, 8 were consistent in the direction of the sex difference and significance in the AusDiab cohort [12]. Similarly, of the 11 subclasses reported in GOLDN to differ by age, 9 were significantly associated with age in the AusDiab cohort [12]. Most interesting, however, is that the relationship between age and the lipidomic measures in GOLDN differed by sex; in one-third (two out of six) of lipid classes, one-third (five out of 15) of lipid subclasses, and about one fourth (97 out of 413) of individual lipid species, age demonstrated either a steeper increase or decline of the lipidomic measure between men and women. Four of the five subclasses reported in GOLDN to exhibit differential age-related associations by sex were also identified in the AusDiab cohort [12].

It is well understood that circulating lipoproteins are affected by female sex steroids, in particular estrogens which demonstrate more robust age-related fluctuations associated with menopause. In the GOLDN study, females had greater age-related increases in both glycerolipids and glycerophospholipids (Table 2, Fig. 1). Among glycerolipids, this sex difference in the relationship of age was also observed in triglycerides but not diglycerides (Table 3). These species are almost exclusively associated with circulating lipoproteins. The present study evaluated the plasma lipidome; further analysis of the lipidome within lipoproteins (LDL, HDL) and their subclasses (HDL1, 2, etc.) may reveal additional insight into the origins of plasma lipids, their potential as biomarkers, and perhaps the impact on aging and disease risk within biological sex.

Galactosylgalactosylceramides (GalGalCer) had an age-related decline in females that was not observed in males (Table 2, Fig. 1). While the plasma source of GalGalCer cannot be discerned from our lipidomic measures, α-galactosidase, a key metabolic enzyme in ceramides, is located on the X chromosome [26]. In addition, messenger RNA for the enzyme has been shown to be significantly repressed in MCF7 breast cancer cells in which estrogen receptor-α had been silenced by siRNAs [27–29]. Consistent with this observation, treatment of breast cancer cell lines with 17β-estradiol has been shown to produce a time-dependent increase in mRNA expression [30, 31]. Collectively, these data suggest a positive correlation between estrogen signaling and α-galactosidase expression that may underlie the age-associated changes in enzyme substrates over the lifespan in females but not in males.

We also found a sex-dependent age-related increase in choline-containing phospholipids, including phosphatidylcholines (PCs) which are the most abundant phospholipids in cellular membranes. Like PCs, PEs also demonstrated an age-related increase in females but not males. PEs are significantly less abundant in biological membranes than their PC counterparts (~ 20–30% if membrane lipids in liver), but they play critical roles in membrane biology and cellular signaling. PEs are asymmetrically distributed in plasma membranes where 80% are confined to the inner leaflet. The loss of that asymmetry is an inflammatory signaling event in a number of tissues, including heart [32]. PEs are also highly enriched in mitochondrial membranes. Thus, changes in their abundance across the lifespan may reflect well-established age-related declines in basal metabolic rate [33]. The most significant PE demonstrating an age*sex interaction was 40:7, which contains an omega – 3 PUFA. Such lipids are well established to influence a number of cardiovascular functions including endothelial and cardiomyocyte function and hemostasis [34].

### Study strengths and limitations

This study has several strengths. First, the large sample size (*n* = 980) includes a robust characterization of underlying health characteristics such as diet, anthropometry, and medical conditions. Additionally, this large cohort spans a wide range of ages (18 to 87 years), offering a comprehensive view across the adult lifespan. Furthermore, all participants were free of lipid-lowering medications. The measurement of participants’ lipid levels in plasma is also a strength of this study. Although serum is more widely used in clinical practice, the coagulation process used to isolate serum can lead to generation or degeneration of lipid species. Plasma generated from fresh blood is considered to be the most similar to the plasma compartment in vivo; therefore, current recommendations for lipidomic analysis favor plasma over serum [13].

Despite these strengths, some limitations do exist. Examination of age associations in this study is limited by the cross-sectional nature of data collection; as such, our results may be used to target specific lipid species or classes in future longitudinal studies examining intra-individual age-related changes. Further, the generalizability of results from this study is limited to similar populations, and these findings should be confirmed in other populations. In particular, participants in the GOLDN cohort predominantly identify as white, so results should only be generalized to populations of European descent.

Residual plots and histograms of residuals were visually examined to assess modeling assumptions of homoscedasticity and normality of residuals for class and subclass analyses. No strong departures from these assumptions were observed, but some minor departures do exist, noted in the glycerolipids class and its two subclasses, TG and DG, as shown in Additional File 3. Because this analysis is a large lipidomic-scale screening phase, we utilized straightforward linear modeling. The intensities of some lipid species, classes, and subclasses may plateau at a certain age, possibly around menopause in women. Our lipidomic screening phase analysis has identified two classes, five subclasses, and 97 individual lipid species that exhibit age- and sex-related associations. We recommend further exploration of these associations in this smaller set of lipids using more complex methodologies that can capture non-linear trends, such as spline-based or change point analyses.

## Conclusion

The results of this study show evidence of age and sex-related associations in several lipid species, classes, subclasses, and lipoproteins. These findings underscore the need for sex stratification when examining age-related changes in the plasma lipidome. Future studies may seek to explore more complex age-related associations and/or associations with disease in a small set of lipid species in order to preserve statistical power; the lipid species or groupings identified in our study should be targeted for this purpose. Our findings add to the understanding of changes in the plasma lipidome across the adult lifespan in both sexes, a feature that can inform advances in precision medicine to manage disease in aging populations.

## Supplementary Information

**Additional file 1. ** Continuation of Table 4. Associations between lipid signal intensity with age, sex, and their interaction (includes all lipid species).

**Additional file 2. ** Correlation between total class intensities.

**Additional file 3. ** Diagnostic plots for regression models of lipid classes and subclasses.

## Data Availability

The data that support the findings of this study are available from the corresponding author upon reasonable request.

## Abbreviations

CE: Cholesteryl ester
DG: Diacylglycerol
GalGalCer: Galactosylgalactosylceramide
GlcCer: Glucosylceramide
GOLDN: Genetics of Lipid-Lowering Drugs and Diet Network (study)
HOMA-IR: Homeostatic model assessment of insulin resistance
LC-MS/MS: Liquid chromatography coupled-mass spectrometry
LCer: Lactosylceramide
LPC: Lysophosphatidylcholine
LPE: Lysophosphatidylethanolamine
MDRD: Modification of Diet in Renal Disease (study)
PC: Phosphatidylcholine
PE: Phospatidylethanolamine
PG: Phosphatidylglycerol
PI: Phosphatidylinositol
SE: Standard error
SERRF: Systematic error removal using random forest
TG: Triglyceride
