# Supplementary material for: Age and sex are associated with the plasma lipidome: findings from the GOLDN study
Source: Lipids Health Dis. 2021 Apr 3;20:30. doi: 10.1186/s12944-021-01456-2 (PMC8019182; doi:10.1186/s12944-021-01456-2)
Supplement: Supplementary file 1 — Additional file 1. Continuation of Table 4. Associations between lipid signal intensity with age, sex, and their interaction (includes all lipid species). [file 12944_2021_1456_MOESM1_ESM.docx]

**Additional File 1.** Continuation of Table 4, Associations Between Lipid Signal Intensity with Age, Sex, and Their Interaction (Includes All Lipid Species)

| Lipid Species | Age β (SE) | Sex β (SE) | Age*Sex Interaction β (SE) |
| --- | --- | --- | --- |
| Acylcarnitine (C10:1) | 0.0052 (0.0029) | 0.0244 (0.0642) | 0.0006 (0.0039) |
| Acylcarnitine (C14:2) | 0.0019 (0.0029) | 0.0258 (0.0642) | -0.0018 (0.0039) |
| Acylcarnitine (C18:3) | 0.0049 (0.0028) | 0.0118 (0.0619) | 0.0018 (0.0038) |
| Acylcarnitine (C8:0) | 0.0020 (0.0029) | 0.1178 (0.0643) | -0.0001 (0.0039) |
| Acylcarnitine (C8:1) | 0.0094 (0.0028)*^b^* | -0.0171 (0.0619) | 0.0024 (0.0038) |
| Acylcarnitine C10:0 | 0.0034 (0.0027) | 0.0840 (0.0612) | -0.0017 (0.0037) |
| Acylcarnitine C12:0 | 0.0020 (0.0028) | -0.0078 (0.0632) | -0.0004 (0.0039) |
| Acylcarnitine C16:0 | 0.0104 (0.0027)*^c^* | -0.2597 (0.0609)*^c^* | 0.0005 (0.0037) |
| Acylcarnitine C18:0 | 0.0161 (0.0027)*^c^* | -0.5599 (0.0594)*^c^* | -0.0011 (0.0036) |
| Acylcarnitine C18:1 | 0.0107 (0.0027)*^c^* | -0.1470 (0.0607)*^a^* | -0.0014 (0.0037) |
| Acylcarnitine C18:2 | 0.0095 (0.0028)*^b^* | -0.4090 (0.0616)*^c^* | -0.0038 (0.0038) |
| CE (16:1) | 0.0063 (0.0027) | 0.4530 (0.0607)*^c^* | 0.0038 (0.0037) |
| CE (18:1) | 0.0074 (0.0028)*^a^* | 0.2702 (0.0613)*^c^* | 0.0015 (0.0037) |
| CE (18:2) | 0.0100 (0.0028)*^b^* | 0.2201 (0.0626)*^b^* | -0.0030 (0.0038) |
| CE (18:3) | 0.0043 (0.0028) | 0.3328 (0.0625)*^c^* | 0.0100 (0.0038)*^a^* |
| CE (20:3) | -0.0018 (0.0028) | 0.3415 (0.0628)*^c^* | 0.0054 (0.0038) |
| CE (20:4) | 0.0032 (0.0028) | 0.2208 (0.0624)*^c^* | 0.0079 (0.0038) |
| CE (20:5) | 0.0079 (0.0028)*^a^* | 0.1190 (0.0627) | 0.0104 (0.0038)*^a^* |
| CE (22:6) | 0.0196 (0.0027)*^c^* | 0.2096 (0.0604)*^b^* | -0.0009 (0.0037) |
| Ceramide (d18:1/23:0) | 0.0156 (0.0027)*^c^* | -0.1153 (0.0595) | 0.0087 (0.0036) |
| Ceramide (d32:1) | 0.0117 (0.0026)*^c^* | 0.1747 (0.0589)*^b^* | 0.0077 (0.0036) |
| Ceramide (d33:1) | 0.0169 (0.0027)*^c^* | 0.0605 (0.0600) | 0.0018 (0.0037) |
| Ceramide (d34:0) | 0.0159 (0.0027)*^c^* | 0.0246 (0.0597) | -0.0051 (0.0036) |
| Ceramide (d34:1) | 0.0216 (0.0026)*^c^* | -0.0046 (0.0572) | 0.0029 (0.0035) |
| Ceramide (d34:2) | 0.0187 (0.0026)*^c^* | 0.3496 (0.0573)*^c^* | 0.0040 (0.0035) |
| Ceramide (d36:1) | 0.0130 (0.0026)*^c^* | 0.0020 (0.0583) | 0.0039 (0.0036) |
| Ceramide (d38:1) | 0.0148 (0.0026)*^c^* | -0.1071 (0.0588) | 0.0058 (0.0036) |
| Ceramide (d39:1) | 0.0133 (0.0027)*^c^* | -0.0482 (0.0598) | 0.0085 (0.0036) |
| Ceramide (d40:0) | 0.0099 (0.0026)*^c^* | -0.0038 (0.0590) | -0.0001 (0.0036) |
| Ceramide (d40:1) | 0.0162 (0.0026)*^c^* | -0.2242 (0.0590)*^c^* | 0.0061 (0.0036) |
| Ceramide (d40:2) | 0.0127 (0.0027)*^c^* | -0.0645 (0.0607) | 0.0053 (0.0037) |
| Ceramide (d41:1) | 0.0136 (0.0027)*^c^* | -0.1077 (0.0594) | 0.0074 (0.0036) |
| Ceramide (d42:0) | 0.0107 (0.0026)*^c^* | -0.0317 (0.0590) | -0.0011 (0.0036) |
| Ceramide (d42:1) | 0.0174 (0.0027)*^c^* | -0.3005 (0.0594)*^c^* | 0.0036 (0.0036) |
| Ceramide (d42:2) | 0.0199 (0.0026)*^c^* | -0.2083 (0.0586)*^c^* | 0.0046 (0.0036) |
| Ceramide (d42:2) A | 0.0203 (0.0026)*^c^* | -0.1762 (0.0587)*^b^* | 0.0024 (0.0036) |
| Ceramide (d42:2) B | 0.0136 (0.0027)*^c^* | -0.1305 (0.0607) | 0.0034 (0.0037) |
| Ceramide (d43:1) | 0.0150 (0.0027)*^c^* | -0.3602 (0.0598)*^c^* | 0.0013 (0.0036) |
| Ceramide (d44:1) | 0.0189 (0.0026)*^c^* | -0.2221 (0.0580)*^c^* | 0.0005 (0.0035) |
| Cholesterol | -0.0123 (0.0027)*^c^* | -0.2907 (0.0595)*^c^* | 0.0012 (0.0036) |
| DG (32:1) | -0.0015 (0.0028) | 0.0064 (0.0616) | 0.0133 (0.0038)*^b^* |
| DG (34:1) | 0.0029 (0.0027) | -0.1502 (0.0609)*^a^* | 0.0087 (0.0037) |
| DG (34:2) | 0.0011 (0.0028) | -0.1062 (0.0615) | 0.0095 (0.0037)*^a^* |
| DG (34:3) | -0.0019 (0.0028) | 0.0796 (0.0616) | 0.0129 (0.0038)*^b^* |
| DG (36:1) | -0.0037 (0.0028) | -0.0625 (0.0618) | 0.0085 (0.0038) |
| DG (36:2) | 0.0048 (0.0027) | -0.2021 (0.0607)*^b^* | 0.0085 (0.0037) |
| DG (36:3) | 0.0054 (0.0028) | -0.1900 (0.0622)*^b^* | 0.0049 (0.0038) |
| DG (36:4) | 0.0050 (0.0028) | -0.1739 (0.0635)*^a^* | 0.0011 (0.0039) |
| DG (36:5) | 0.0010 (0.0028) | -0.0376 (0.0634) | 0.0084 (0.0039) |
| DG (38:0) | 0.0142 (0.0026)*^c^* | -0.1300 (0.0587)*^a^* | 0.0049 (0.0036) |
| DG (38:2) | 0.0008 (0.0029) | -0.1555 (0.0638)*^a^* | -0.0004 (0.0039) |
| DG (38:3) | 0.0028 (0.0028) | -0.0017 (0.0631) | 0.0054 (0.0038) |
| DG (38:5) | 0.0028 (0.0028) | -0.0564 (0.0616) | 0.0129 (0.0038)*^b^* |
| DG (38:6) A | 0.0036 (0.0028) | -0.0689 (0.0626) | 0.0088 (0.0038) |
| DG (38:6) B | 0.0039 (0.0028) | -0.0720 (0.0630) | 0.0086 (0.0038) |
| DG (40:6) | 0.0080 (0.0028)*^a^* | -0.0133 (0.0630) | 0.0012 (0.0038) |
| DG (40:7) | 0.0069 (0.0028)*^a^* | -0.0483 (0.0614) | 0.0086 (0.0037) |
| FA (10:0) (capric acid) | 0.0009 (0.0028) | 0.1306 (0.0614) | 0.0010 (0.0037) |
| FA (12:0) | 0.0018 (0.0027) | 0.3104 (0.0604)*^c^* | -0.0026 (0.0037) |
| FA (13:0) | 0.0002 (0.0028) | 0.1251 (0.0622) | -0.0046 (0.0038) |
| FA (14:0) (myristic acid) | 0.0010 (0.0027) | 0.4183 (0.0607)*^c^* | 0.0031 (0.0037) |
| FA (14:1) | 0.0005 (0.0027) | 0.5072 (0.0609)*^c^* | -0.0013 (0.0037) |
| FA (15:0) A | 0.0018 (0.0028) | 0.1946 (0.0635)*^b^* | -0.0003 (0.0039) |
| FA (15:0) B | -0.0017 (0.0028) | 0.2113 (0.0614)*^b^* | 0.0033 (0.0037) |
| FA (15:1) | 0.0001 (0.0028) | 0.1105 (0.0620) | 0.0031 (0.0038) |
| FA (16:0) | 0.0042 (0.0028) | 0.2402 (0.0617)*^c^* | -0.0030 (0.0038) |
| FA (16:1) | 0.0032 (0.0026) | 0.6139 (0.0590)*^c^* | 0.0027 (0.0036) |
| FA (17:1) (heptadecenoic acid) | 0.0019 (0.0027) | 0.3890 (0.0600)*^c^* | 0.0050 (0.0037) |
| FA (18:0) | 0.0023 (0.0027) | 0.0308 (0.0593) | -0.0010 (0.0036) |
| FA (18:1) | 0.0074 (0.0027)*^a^* | 0.2853 (0.0596)*^c^* | -0.0019 (0.0036) |
| FA (18:2) | 0.0074 (0.0027)*^a^* | 0.2893 (0.0605)*^c^* | -0.0023 (0.0037) |
| FA (18:3) | 0.0096 (0.0027)*^b^* | 0.3929 (0.0596)*^c^* | 0.0001 (0.0036) |
| FA (19:0) | -0.0006 (0.0024) | -0.0210 (0.0544) | 0.0021 (0.0033) |
| FA (20:0) (arachidic acid) | -0.0003 (0.0027) | -0.0238 (0.0597) | 0.0005 (0.0036) |
| FA (20:1) (eicosenoic acid) | 0.0091 (0.0028)*^b^* | 0.1153 (0.0618) | -0.0020 (0.0038) |
| FA (20:2) (eicosadienoic acid) | 0.0065 (0.0027) | 0.2148 (0.0611)*^b^* | -0.0051 (0.0037) |
| FA (20:3) (homo-gamma-linolenic acid) | 0.0042 (0.0028) | 0.0505 (0.0615) | 0.0034 (0.0037) |
| FA (20:4) | 0.0055 (0.0028) | -0.1044 (0.0627) | 0.0034 (0.0038) |
| FA (20:5) | -0.0017 (0.0029) | 0.0537 (0.0639) | 0.0022 (0.0039) |
| FA (20:6) | -0.0015 (0.0027) | 0.0066 (0.0608) | 0.0027 (0.0037) |
| FA (22:0) (behenic acid) | -0.0017 (0.0028) | -0.1430 (0.0634)*^a^* | 0.0027 (0.0039) |
| FA (22:1) (erucic acid) | 0.0052 (0.0029) | -0.0608 (0.0639) | -0.0064 (0.0039) |
| FA (22:4) | 0.0027 (0.0027) | 0.1846 (0.0603)*^b^* | 0.0038 (0.0037) |
| FA (22:5) (docosapentaenoic acid) | 0.0060 (0.0027) | 0.1192 (0.0602) | 0.0052 (0.0037) |
| FA (22:6) | 0.0032 (0.0028) | 0.0355 (0.0629) | -0.0042 (0.0038) |
| FA (24:0) (lignoceric acid) | 0.0012 (0.0028) | -0.0138 (0.0625) | 0.0074 (0.0038) |
| FA (24:1) (nervonic acid) | 0.0106 (0.0027)*^c^* | -0.2368 (0.0607)*^c^* | -0.0034 (0.0037) |
| FA (26:0) (cerotic acid) | -0.0011 (0.0028) | -0.0551 (0.0627) | 0.0056 (0.0038) |
| FA (28:0) (montanic acid) | -0.0019 (0.0028) | -0.0277 (0.0614) | 0.0031 (0.0037) |
| GalGalCer (d18:1/16:0) or LCer (d18:1/16:0) | 0.0000 (0.0027) | -0.0223 (0.0602) | -0.0130 (0.0037)*^b^* |
| GlcCer (d38:1) | 0.0170 (0.0027)*^c^* | 0.2187 (0.0609)*^c^* | 0.0012 (0.0037) |
| GlcCer (d40:1) | 0.0130 (0.0028)*^c^* | -0.1555 (0.0623)*^a^* | -0.0025 (0.0038) |
| GlcCer (d41:1) | 0.0089 (0.0028)*^b^* | -0.0578 (0.0626) | 0.0004 (0.0038) |
| GlcCer (d42:1) | 0.0121 (0.0028)*^c^* | -0.2703 (0.0618)*^c^* | -0.0007 (0.0038) |
| GlcCer (d42:2) | 0.0127 (0.0028)*^c^* | 0.1698 (0.0623)*^a^* | -0.0025 (0.0038) |
| GlcCer(d14:1(4E)/20:0(2OH)) | 0.0090 (0.0021)*^c^* | 0.1474 (0.0466)*^b^* | 0.0069 (0.0028) |
| LCer (d18:1/24:1(15Z)) | 0.0109 (0.0028)*^c^* | 0.0242 (0.0624) | -0.0008 (0.0038) |
| LPC (14:0) | -0.0012 (0.0028) | -0.0229 (0.0631) | 0.0089 (0.0038) |
| LPC (15:0) | 0.0002 (0.0028) | -0.3165 (0.0627)*^c^* | 0.0101 (0.0038)*^a^* |
| LPC (16:0) | 0.0073 (0.0028)*^a^* | -0.4020 (0.0618)*^c^* | 0.0067 (0.0038) |
| LPC (16:1) | 0.0039 (0.0028) | -0.1170 (0.0632) | 0.0060 (0.0039) |
| LPC (17:1) | -0.0002 (0.0027) | -0.5260 (0.0612)*^c^* | 0.0118 (0.0037)*^a^* |
| LPC (18:0) | 0.0056 (0.0028) | -0.3528 (0.0620)*^c^* | 0.0105 (0.0038)*^a^* |
| LPC (18:0) A | 0.0061 (0.0028) | -0.3750 (0.0616)*^c^* | 0.0084 (0.0038) |
| LPC (18:1) | 0.0019 (0.0027) | -0.5462 (0.0595)*^c^* | 0.0087 (0.0036) |
| LPC (18:2) | -0.0122 (0.0026)*^c^* | -0.5867 (0.0575)*^c^* | 0.0067 (0.0035) |
| LPC (18:3) | -0.0033 (0.0028) | -0.2860 (0.0613)*^c^* | 0.0085 (0.0037) |
| LPC (20:0) | 0.0083 (0.0027)*^b^* | -0.3504 (0.0609)*^c^* | 0.0029 (0.0037) |
| LPC (20:1) | 0.0113 (0.0027)*^c^* | -0.3796 (0.0600)*^c^* | 0.0023 (0.0037) |
| LPC (20:2) | 0.0046 (0.0028) | -0.3869 (0.0617)*^c^* | 0.0044 (0.0038) |
| LPC (20:3) | -0.0086 (0.0028)*^b^* | -0.5081 (0.0619)*^c^* | 0.0142 (0.0038)*^b^* |
| LPC (20:4) | -0.0070 (0.0027)*^a^* | -0.5721 (0.0603)*^c^* | 0.0134 (0.0037)*^b^* |
| LPC (20:5) | -0.0039 (0.0029) | -0.1202 (0.0637) | 0.0055 (0.0039) |
| LPC (22:4) | -0.0083 (0.0028)*^b^* | -0.4874 (0.0614)*^c^* | 0.0114 (0.0037)*^a^* |
| LPC (22:5) | -0.0049 (0.0027) | -0.5314 (0.0609)*^c^* | 0.0146 (0.0037)*^b^* |
| LPC (22:6) | 0.0106 (0.0027)*^c^* | -0.3391 (0.0612)*^c^* | 0.0047 (0.0037) |
| LPC (o-16:0) | 0.0049 (0.0028) | -0.4947 (0.0615)*^c^* | 0.0033 (0.0037) |
| LPC (p-16:0) or LPC (o-16:1) | 0.0030 (0.0027) | -0.5718 (0.0607)*^c^* | 0.0091 (0.0037) |
| LPC (p-18:0) or LPC (o-18:1) | 0.0116 (0.0027)*^c^* | -0.3747 (0.0612)*^c^* | 0.0024 (0.0037) |
| LPE (16:0) | 0.0116 (0.0028)*^c^* | -0.0684 (0.0621) | 0.0041 (0.0038) |
| LPE (18:0) | 0.0072 (0.0028)*^a^* | -0.0927 (0.0616) | 0.0072 (0.0038) |
| LPE (18:2) | -0.0039 (0.0028) | -0.2067 (0.0622)*^b^* | 0.0023 (0.0038) |
| LPE (20:4) | -0.0034 (0.0028) | -0.2305 (0.0620)*^c^* | 0.0076 (0.0038) |
| LPE (22:6) | 0.0156 (0.0027)*^c^* | 0.1881 (0.0595)*^b^* | 0.0030 (0.0036) |
| PC (16:0/9:0(CHO)) | -0.0001 (0.0027) | 0.1243 (0.0611) | -0.0009 (0.0037) |
| PC (28:0) | -0.0008 (0.0029) | 0.2423 (0.0638)*^c^* | 0.0087 (0.0039) |
| PC (30:0) | 0.0037 (0.0028) | 0.3825 (0.0619)*^c^* | 0.0100 (0.0038)*^a^* |
| PC (30:1) | 0.0000 (0.0028) | 0.4766 (0.0618)*^c^* | 0.0113 (0.0038)*^a^* |
| PC (31:0) | 0.0095 (0.0027)*^b^* | 0.4353 (0.0605)*^c^* | 0.0084 (0.0037) |
| PC (31:1) | 0.0023 (0.0027) | 0.4413 (0.0605)*^c^* | 0.0099 (0.0037)*^a^* |
| PC (32:0) | 0.0139 (0.0027)*^c^* | 0.1683 (0.0610)*^a^* | 0.0000 (0.0037) |
| PC (32:1) | 0.0026 (0.0026) | 0.1600 (0.0569)*^b^* | 0.0023 (0.0035) |
| PC (32:2) | 0.0003 (0.0027) | 0.6422 (0.0602)*^c^* | 0.0104 (0.0037)*^a^* |
| PC (32:3) | 0.0039 (0.0028) | 0.3742 (0.0614)*^c^* | 0.0101 (0.0037)*^a^* |
| PC (33:0) | 0.0072 (0.0027)*^a^* | 0.1736 (0.0612)*^b^* | 0.0121 (0.0037)*^a^* |
| PC (33:1) | 0.0022 (0.0028) | 0.1189 (0.0634) | 0.0072 (0.0039) |
| PC (33:2) | -0.0061 (0.0028) | 0.3649 (0.0617)*^c^* | 0.0043 (0.0038) |
| PC (34:0) | 0.0152 (0.0026)*^c^* | -0.2188 (0.0588)*^c^* | 0.0018 (0.0036) |
| PC (34:1) | 0.0109 (0.0028)*^c^* | 0.1886 (0.0616)*^b^* | 0.0031 (0.0038) |
| PC (34:2) | 0.0098 (0.0027)*^b^* | 0.3457 (0.0603)*^c^* | 0.0004 (0.0037) |
| PC (34:3) | 0.0082 (0.0027)*^b^* | 0.6636 (0.0593)*^c^* | 0.0083 (0.0036) |
| PC (34:3) A | 0.0000 (0.0027) | 0.3695 (0.0604)*^c^* | 0.0028 (0.0037) |
| PC (34:3) B | 0.0069 (0.0026)*^a^* | 0.4280 (0.0576)*^c^* | -0.0030 (0.0035) |
| PC (34:4) | -0.0022 (0.0027) | 0.5713 (0.0598)*^c^* | 0.0163 (0.0036)*^c^* |
| PC (35:1) | 0.0103 (0.0028)*^b^* | -0.1158 (0.0615) | 0.0014 (0.0037) |
| PC (35:2) | 0.0038 (0.0027) | 0.0670 (0.0601) | 0.0022 (0.0037) |
| PC (35:2) B | 0.0113 (0.0028)*^c^* | 0.2264 (0.0616)*^c^* | 0.0043 (0.0038) |
| PC (35:3) | 0.0063 (0.0028) | 0.3645 (0.0614)*^c^* | 0.0096 (0.0037)*^a^* |
| PC (35:4) | 0.0006 (0.0027) | 0.4934 (0.0608)*^c^* | 0.0156 (0.0037)*^b^* |
| PC (36:1) | 0.0066 (0.0028)*^a^* | 0.1112 (0.0620) | 0.0076 (0.0038) |
| PC (36:2) | 0.0055 (0.0028) | 0.2523 (0.0616)*^c^* | 0.0071 (0.0038) |
| PC (36:3) A | 0.0018 (0.0027) | 0.2081 (0.0611)*^b^* | 0.0118 (0.0037)*^a^* |
| PC (36:3) B | 0.0015 (0.0020) | 0.0829 (0.0438) | -0.0016 (0.0027) |
| PC (36:4) A | -0.0037 (0.0028) | 0.0125 (0.0622) | -0.0005 (0.0038) |
| PC (36:4) B | 0.0017 (0.0028) | 0.1802 (0.0620)*^b^* | 0.0061 (0.0038) |
| PC (36:4) C | 0.0123 (0.0025)*^c^* | 0.1257 (0.0559)*^a^* | 0.0032 (0.0034) |
| PC (36:5) A | 0.0026 (0.0028) | 0.2409 (0.0627)*^c^* | 0.0009 (0.0038) |
| PC (36:5) B | 0.0082 (0.0028)*^a^* | 0.1680 (0.0618)*^a^* | 0.0109 (0.0038)*^a^* |
| PC (36:5) C | 0.0022 (0.0029) | -0.0720 (0.0637) | 0.0023 (0.0039) |
| PC (36:6) | 0.0074 (0.0026)*^a^* | 0.5758 (0.0588)*^c^* | 0.0130 (0.0036)*^b^* |
| PC (37:2) | 0.0071 (0.0028)*^a^* | 0.2769 (0.0619)*^c^* | 0.0072 (0.0038) |
| PC (37:3) | 0.0019 (0.0025) | 0.1764 (0.0548)*^b^* | 0.0099 (0.0033)*^a^* |
| PC (37:4) | 0.0018 (0.0024) | 0.0480 (0.0534) | 0.0043 (0.0033) |
| PC (37:5) | 0.0069 (0.0028)*^a^* | 0.1583 (0.0615)*^a^* | 0.0132 (0.0037)*^b^* |
| PC (37:6) | 0.0116 (0.0027)*^c^* | 0.4078 (0.0602)*^c^* | 0.0080 (0.0037) |
| PC (38:2) | 0.0119 (0.0027)*^c^* | 0.4051 (0.0601)*^c^* | 0.0034 (0.0037) |
| PC (38:3) | -0.0004 (0.0028) | 0.0853 (0.0616) | 0.0108 (0.0038)*^a^* |
| PC (38:4) | -0.0018 (0.0027) | 0.1720 (0.0611)*^b^* | 0.0169 (0.0037)*^c^* |
| PC (38:4) A | 0.0026 (0.0027) | 0.2269 (0.0595)*^c^* | 0.0192 (0.0036)*^c^* |
| PC (38:4) B | 0.0089 (0.0027)*^b^* | 0.3144 (0.0599)*^c^* | 0.0136 (0.0036)*^b^* |
| PC (38:5) A | 0.0085 (0.0027)*^b^* | 0.0393 (0.0600) | 0.0126 (0.0037)*^b^* |
| PC (38:5) B | 0.0002 (0.0028) | 0.2053 (0.0629)*^b^* | 0.0123 (0.0038)*^a^* |
| PC (38:6) | 0.0178 (0.0025)*^c^* | 0.2270 (0.0561)*^c^* | 0.0043 (0.0034) |
| PC (38:6) A | 0.0209 (0.0026)*^c^* | 0.2980 (0.0582)*^c^* | 0.0047 (0.0035) |
| PC (38:6) B | 0.0044 (0.0022) | 0.3453 (0.0485)*^c^* | 0.0046 (0.0030) |
| PC (38:6) C | 0.0171 (0.0026)*^c^* | 0.2204 (0.0575)*^c^* | 0.0050 (0.0035) |
| PC (38:7) | 0.0071 (0.0028)*^a^* | 0.2256 (0.0628)*^c^* | 0.0062 (0.0038) |
| PC (39:6) | 0.0176 (0.0027)*^c^* | 0.2171 (0.0597)*^c^* | 0.0061 (0.0036) |
| PC (40:4) | -0.0026 (0.0028) | 0.0449 (0.0620) | 0.0140 (0.0038)*^b^* |
| PC (40:5) A | 0.0055 (0.0028) | -0.1127 (0.0614) | 0.0131 (0.0037)*^b^* |
| PC (40:5) B | -0.0012 (0.0028) | 0.2397 (0.0628)*^c^* | 0.0018 (0.0038) |
| PC (40:6) A | 0.0043 (0.0029) | -0.0219 (0.0637) | -0.0030 (0.0039) |
| PC (40:6) B | 0.0206 (0.0026)*^c^* | 0.1999 (0.0587)*^b^* | 0.0029 (0.0036) |
| PC (40:7) | 0.0189 (0.0026)*^c^* | 0.2285 (0.0590)*^c^* | 0.0012 (0.0036) |
| PC (40:8) | 0.0096 (0.0027)*^b^* | 0.0403 (0.0600) | 0.0099 (0.0037)*^a^* |
| PC (42:10) | 0.0157 (0.0026)*^c^* | 0.1568 (0.0590)*^a^* | 0.0108 (0.0036)*^a^* |
| PC (42:5) | 0.0087 (0.0027)*^b^* | 0.4267 (0.0604)*^c^* | 0.0085 (0.0037) |
| PC (42:6) | 0.0002 (0.0028) | 0.2738 (0.0614)*^c^* | 0.0120 (0.0037)*^a^* |
| PC (42:7) | 0.0175 (0.0027)*^c^* | 0.3824 (0.0598)*^c^* | -0.0009 (0.0036) |
| PC (o-32:0) | 0.0024 (0.0028) | 0.0584 (0.0616) | -0.0059 (0.0038) |
| PC (o-34:0) | 0.0109 (0.0028)*^c^* | 0.0444 (0.0615) | -0.0038 (0.0037) |
| PC (p-32:0) or PC (o-32:1) | 0.0097 (0.0028)*^b^* | -0.0395 (0.0626) | -0.0008 (0.0038) |
| PC (p-32:1) or PC (o-32:2) | 0.0143 (0.0027)*^c^* | 0.2388 (0.0606)*^c^* | 0.0082 (0.0037) |
| PC (p-34:0) or PC (o-34:1) | 0.0121 (0.0027)*^c^* | 0.2579 (0.0611)*^c^* | -0.0055 (0.0037) |
| PC (p-34:1) or PC (o-34:2) A | 0.0011 (0.0028) | -0.0433 (0.0624) | -0.0048 (0.0038) |
| PC (p-34:1) or PC (o-34:2) B | 0.0120 (0.0027)*^c^* | 0.3771 (0.0594)*^c^* | -0.0020 (0.0036) |
| PC (p-34:2) or PC (o-34:3) | -0.0017 (0.0028) | 0.0509 (0.0624) | -0.0014 (0.0038) |
| PC (p-36:1) or PC (o-36:2) | 0.0021 (0.0027) | 0.0892 (0.0601) | -0.0098 (0.0037)*^a^* |
| PC (p-36:1) or PC (o-36:2) A | 0.0140 (0.0026)*^c^* | 0.2617 (0.0581)*^c^* | 0.0060 (0.0035) |
| PC (p-36:2) or PC (o-36:3) | 0.0037 (0.0028) | 0.0262 (0.0630) | -0.0061 (0.0038) |
| PC (p-36:3) or PC (o-36:4) | -0.0015 (0.0027) | -0.4082 (0.0609)*^c^* | 0.0035 (0.0037) |
| PC (p-36:4) or PC (o-36:5) | 0.0002 (0.0028) | -0.3468 (0.0621)*^c^* | 0.0092 (0.0038) |
| PC (p-38:2) or PC (o-38:3) | 0.0000 (0.0028) | 0.1812 (0.0631)*^b^* | -0.0020 (0.0038) |
| PC (p-38:3) or PC (o-38:4) | -0.0033 (0.0028) | -0.2496 (0.0613)*^c^* | -0.0038 (0.0037) |
| PC (p-38:3) or PC (o-38:4) B | -0.0010 (0.0028) | -0.1117 (0.0630) | 0.0012 (0.0038) |
| PC (p-38:4) or PC (o-38:5) A | 0.0037 (0.0028) | -0.2081 (0.0617)*^b^* | 0.0047 (0.0038) |
| PC (p-38:4) or PC (o-38:5) B | 0.0020 (0.0028) | -0.3457 (0.0617)*^c^* | 0.0064 (0.0038) |
| PC (p-38:5) or PC (o-38:6) | 0.0007 (0.0028) | -0.0806 (0.0631) | 0.0060 (0.0038) |
| PC (p-38:5) or PC (o-38:6) A | 0.0071 (0.0026)*^a^* | 0.2561 (0.0587)*^c^* | 0.0201 (0.0036)*^c^* |
| PC (p-38:6) or PC (o-38:7) | 0.0175 (0.0027)*^c^* | 0.0426 (0.0599) | 0.0076 (0.0037) |
| PC (p-40:1) or PC (o-40:2) | 0.0043 (0.0028) | 0.2920 (0.0619)*^c^* | -0.0046 (0.0038) |
| PC (p-40:3) or PC (o-40:4) | 0.0010 (0.0028) | 0.0972 (0.0631) | -0.0071 (0.0038) |
| PC (p-40:4) or PC (o-40:5) | 0.0046 (0.0028) | 0.0942 (0.0613) | 0.0013 (0.0037) |
| PC (p-40:5) or PC (o-40:6) | 0.0147 (0.0027)*^c^* | 0.2620 (0.0605)*^c^* | 0.0041 (0.0037) |
| PC (p-40:6) or PC (o-40:7) A | 0.0207 (0.0026)*^c^* | 0.2571 (0.0588)*^c^* | 0.0030 (0.0036) |
| PC (p-40:6) or PC (o-40:7) B | 0.0174 (0.0027)*^c^* | 0.1117 (0.0606) | 0.0043 (0.0037) |
| PC (p-40:7) or PC (o-40:8) | 0.0114 (0.0027)*^c^* | 0.2293 (0.0607)*^c^* | 0.0037 (0.0037) |
| PC (p-42:2) or PC (o-42:3) | 0.0059 (0.0027) | 0.3109 (0.0604)*^c^* | -0.0094 (0.0037)*^a^* |
| PC (p-42:3) or PC (o-42:4) | 0.0053 (0.0028) | 0.0526 (0.0633) | -0.0060 (0.0039) |
| PC (p-42:4) or PC (o-42:5) | 0.0031 (0.0028) | 0.3205 (0.0624)*^c^* | -0.0013 (0.0038) |
| PC (p-42:5) or PC (o-42:6) | -0.0012 (0.0028) | 0.1446 (0.0616)*^a^* | -0.0086 (0.0038) |
| PC (p-42:5) or PC (o-42:6) A | 0.0074 (0.0024)*^a^* | 0.0848 (0.0544) | -0.0058 (0.0033) |
| PC (p-44:4) or PC (o-44:5) | 0.0073 (0.0028)*^a^* | 0.2114 (0.0628)*^b^* | -0.0005 (0.0038) |
| PC (p-44:5) or PC (o-44:6) | 0.0059 (0.0027) | 0.0477 (0.0593) | -0.0002 (0.0036) |
| PE (32:1) | 0.0018 (0.0028) | 0.3792 (0.0614)*^c^* | 0.0069 (0.0037) |
| PE (34:1) | 0.0055 (0.0027) | 0.3584 (0.0609)*^c^* | 0.0055 (0.0037) |
| PE (34:2) | 0.0001 (0.0028) | 0.2184 (0.0616)*^c^* | 0.0031 (0.0038) |
| PE (34:3) | 0.0043 (0.0027) | 0.2278 (0.0606)*^c^* | 0.0004 (0.0037) |
| PE (36:1) | 0.0035 (0.0028) | 0.1464 (0.0619)*^a^* | 0.0055 (0.0038) |
| PE (36:2) | 0.0029 (0.0027) | 0.3022 (0.0595)*^c^* | 0.0080 (0.0036) |
| PE (36:3) | 0.0048 (0.0027) | 0.3674 (0.0606)*^c^* | 0.0022 (0.0037) |
| PE (36:4) | 0.0002 (0.0027) | 0.2381 (0.0597)*^c^* | 0.0075 (0.0036) |
| PE (38:2) | 0.0054 (0.0028) | 0.2154 (0.0616)*^b^* | 0.0054 (0.0038) |
| PE (38:4) | 0.0026 (0.0026) | 0.3071 (0.0586)*^c^* | 0.0109 (0.0036)*^a^* |
| PE (38:4) A | 0.0020 (0.0027) | 0.1813 (0.0612)*^b^* | 0.0085 (0.0037) |
| PE (38:4) B | 0.0029 (0.0027) | 0.3247 (0.0593)*^c^* | 0.0111 (0.0036)*^a^* |
| PE (38:6) | 0.0090 (0.0026)*^b^* | 0.5543 (0.0588)*^c^* | 0.0101 (0.0036)*^a^* |
| PE (38:7) | 0.0035 (0.0026) | 0.3871 (0.0575)*^c^* | 0.0053 (0.0035) |
| PE (40:6) | 0.0013 (0.0028) | 0.1326 (0.0627) | 0.0045 (0.0038) |
| PE (40:7) | 0.0015 (0.0027) | 0.3818 (0.0607)*^c^* | 0.0127 (0.0037)*^b^* |
| PE (40:8) | -0.0036 (0.0027) | 0.1167 (0.0602) | 0.0013 (0.0037) |
| PE (44:3) | 0.0031 (0.0027) | -0.0593 (0.0600) | 0.0015 (0.0037) |
| PE (p-34:1) or PE (o-34:2) | 0.0054 (0.0028) | -0.0307 (0.0616) | 0.0099 (0.0038)*^a^* |
| PE (p-34:2) or PE (o-34:3) | 0.0077 (0.0026)*^a^* | -0.0333 (0.0577) | 0.0051 (0.0035) |
| PE (p-36:1) or PE (o-36:2) | 0.0067 (0.0028)*^a^* | -0.0978 (0.0623) | 0.0054 (0.0038) |
| PE (p-36:2) or PE (o-36:3) | 0.0072 (0.0028)*^a^* | -0.0473 (0.0613) | 0.0075 (0.0037) |
| PE (p-36:4) or PE (o-36:5) | 0.0035 (0.0027) | -0.1531 (0.0603)*^a^* | 0.0104 (0.0037)*^a^* |
| PE (p-36:5) or PE (o-36:6) | 0.0044 (0.0028) | 0.0082 (0.0613) | 0.0102 (0.0037)*^a^* |
| PE (p-38:2) or PE (o-38:3) | 0.0088 (0.0028)*^b^* | 0.0439 (0.0618) | 0.0031 (0.0038) |
| PE (p-38:3) or PE (o-38:4) | 0.0041 (0.0028) | 0.0233 (0.0618) | 0.0073 (0.0038) |
| PE (p-38:4) or PE (o-38:5) | 0.0025 (0.0025) | -0.0468 (0.0553) | 0.0042 (0.0034) |
| PE (p-38:5) or PE (o-38:6) | 0.0036 (0.0026) | -0.0775 (0.0587) | 0.0111 (0.0036)*^a^* |
| PE (p-38:6) or PE (o-38:7) | 0.0117 (0.0025)*^c^* | 0.2097 (0.0547)*^c^* | 0.0070 (0.0033) |
| PE (p-40:4) or PE (o-40:5) | 0.0045 (0.0027) | -0.0161 (0.0597) | 0.0074 (0.0036) |
| PE (p-40:4) or PE (o-40:5) A | 0.0013 (0.0023) | -0.0017 (0.0513) | 0.0040 (0.0031) |
| PE (p-40:4) or PE (o-40:5) B | -0.0012 (0.0025) | -0.0552 (0.0566) | 0.0015 (0.0034) |
| PE (p-40:5) or PE (o-40:6) | 0.0002 (0.0027) | -0.0985 (0.0611) | 0.0042 (0.0037) |
| PE (p-40:6) or PE (o-40:7) | 0.0132 (0.0025)*^c^* | 0.1065 (0.0558) | 0.0077 (0.0034) |
| PE (p-40:7) or PE (o-40:8) | 0.0123 (0.0025)*^c^* | 0.2364 (0.0563)*^c^* | 0.0097 (0.0034)*^a^* |
| PG (34:2) A | -0.0002 (0.0028) | 0.2538 (0.0635)*^c^* | 0.0008 (0.0039) |
| PG (34:2) B | 0.0090 (0.0025)*^b^* | 0.1189 (0.0559) | 0.0046 (0.0034) |
| PG (36:4) | -0.0007 (0.0028) | 0.0293 (0.0629) | -0.0014 (0.0038) |
| PG (38:7) | 0.0058 (0.0027) | 0.3825 (0.0597)*^c^* | 0.0037 (0.0036) |
| PG (40:7) | 0.0071 (0.0027)*^a^* | 0.2073 (0.0611)*^b^* | -0.0006 (0.0037) |
| PG (40:8) | 0.0013 (0.0029) | -0.0244 (0.0635) | -0.0025 (0.0039) |
| PG (44:12) | 0.0033 (0.0029) | 0.0004 (0.0636) | -0.0007 (0.0039) |
| PI (32:1) | 0.0005 (0.0028) | 0.3584 (0.0615)*^c^* | 0.0051 (0.0037) |
| PI (34:1) | 0.0035 (0.0028) | 0.3945 (0.0620)*^c^* | 0.0024 (0.0038) |
| PI (34:2) | 0.0032 (0.0027) | 0.4388 (0.0611)*^c^* | 0.0018 (0.0037) |
| PI (36:1) | 0.0039 (0.0027) | 0.3506 (0.0612)*^c^* | 0.0012 (0.0037) |
| PI (36:2) | 0.0061 (0.0028) | 0.5028 (0.0616)*^c^* | -0.0036 (0.0038) |
| PI (36:3) | 0.0042 (0.0028) | 0.4384 (0.0620)*^c^* | -0.0025 (0.0038) |
| PI (36:4) | 0.0040 (0.0027) | 0.2849 (0.0611)*^c^* | 0.0093 (0.0037) |
| PI (38:3) | 0.0032 (0.0025) | 0.1014 (0.0551) | 0.0076 (0.0034) |
| PI (38:4) | 0.0077 (0.0027)*^a^* | 0.0448 (0.0609) | 0.0080 (0.0037) |
| PI (38:5) | 0.0040 (0.0028) | 0.2493 (0.0623)*^c^* | 0.0058 (0.0038) |
| PI (38:6) | 0.0112 (0.0027)*^c^* | 0.4842 (0.0607)*^c^* | 0.0015 (0.0037) |
| PI (40:6) | 0.0141 (0.0027)*^c^* | 0.4657 (0.0607)*^c^* | 0.0005 (0.0037) |
| SM (d30:1) | 0.0029 (0.0026) | 0.7290 (0.0580)*^c^* | 0.0140 (0.0035)*^b^* |
| SM (d32:0) | 0.0121 (0.0027)*^c^* | 0.4503 (0.0604)*^c^* | 0.0017 (0.0037) |
| SM (d32:1) | 0.0129 (0.0026)*^c^* | 0.4154 (0.0579)*^c^* | 0.0125 (0.0035)*^b^* |
| SM (d32:2) | 0.0048 (0.0022) | 1.0661 (0.0485)*^c^* | 0.0131 (0.0030)*^c^* |
| SM (d33:1) | 0.0162 (0.0026)*^c^* | 0.3064 (0.0590)*^c^* | 0.0094 (0.0036)*^a^* |
| SM (d34:0) | 0.0167 (0.0027)*^c^* | 0.3043 (0.0592)*^c^* | 0.0009 (0.0036) |
| SM (d34:1) | 0.0143 (0.0028)*^c^* | 0.1020 (0.0614) | -0.0028 (0.0037) |
| SM (d34:2) | 0.0174 (0.0024)*^c^* | 0.6265 (0.0527)*^c^* | 0.0125 (0.0032)*^b^* |
| SM (d36:0) | 0.0048 (0.0026) | 0.0757 (0.0579) | 0.0001 (0.0035) |
| SM (d36:1) | 0.0072 (0.0027)*^a^* | 0.2372 (0.0609)*^c^* | -0.0013 (0.0037) |
| SM (d36:2) | 0.0096 (0.0027)*^b^* | 0.2917 (0.0603)*^c^* | 0.0085 (0.0037) |
| SM (d36:3) | 0.0005 (0.0027) | 0.6045 (0.0606)*^c^* | 0.0061 (0.0037) |
| SM (d37:1) | 0.0079 (0.0028)*^a^* | 0.2314 (0.0622)*^c^* | 0.0018 (0.0038) |
| SM (d38:0) | 0.0052 (0.0027) | 0.1414 (0.0598)*^a^* | 0.0030 (0.0036) |
| SM (d38:1) | 0.0100 (0.0027)*^b^* | 0.3059 (0.0600)*^c^* | 0.0110 (0.0037)*^a^* |
| SM (d38:2) | 0.0051 (0.0027) | 0.5769 (0.0602)*^c^* | -0.0003 (0.0037) |
| SM (d39:1) | 0.0070 (0.0027)*^a^* | 0.3700 (0.0606)*^c^* | 0.0133 (0.0037)*^b^* |
| SM (d39:2) | 0.0080 (0.0027)*^a^* | 0.6670 (0.0591)*^c^* | 0.0014 (0.0036) |
| SM (d40:0) | 0.0011 (0.0025) | -0.0176 (0.0555) | 0.0010 (0.0034) |
| SM (d40:1) | 0.0098 (0.0027)*^b^* | 0.0792 (0.0607) | 0.0083 (0.0037) |
| SM (d40:2) A | 0.0090 (0.0027)*^b^* | 0.4260 (0.0593)*^c^* | 0.0141 (0.0036)*^b^* |
| SM (d40:2) B | 0.0026 (0.0028) | 0.3104 (0.0617)*^c^* | 0.0002 (0.0038) |
| SM (d40:3) | 0.0025 (0.0027) | 0.5343 (0.0608)*^c^* | -0.0063 (0.0037) |
| SM (d41:1) | 0.0059 (0.0027) | 0.2687 (0.0609)*^c^* | 0.0142 (0.0037)*^b^* |
| SM (d41:2) | 0.0041 (0.0027) | 0.5911 (0.0595)*^c^* | 0.0091 (0.0036) |
| SM (d41:2) A | 0.0032 (0.0028) | 0.2540 (0.0614)*^c^* | 0.0040 (0.0037) |
| SM (d41:2) B | 0.0063 (0.0026)*^a^* | 0.6994 (0.0581)*^c^* | 0.0157 (0.0035)*^c^* |
| SM (d42:0) | 0.0001 (0.0025) | -0.0538 (0.0566) | 0.0023 (0.0035) |
| SM (d42:1) | 0.0080 (0.0028)*^a^* | -0.1564 (0.0614)*^a^* | 0.0069 (0.0037) |
| SM (d42:2) A | 0.0163 (0.0027)*^c^* | 0.2311 (0.0597)*^c^* | 0.0075 (0.0036) |
| SM (d42:2) B | 0.0083 (0.0026)*^b^* | 0.1794 (0.0580)*^b^* | 0.0038 (0.0035) |
| SM (d42:3) | 0.0139 (0.0025)*^c^* | 0.6397 (0.0568)*^c^* | 0.0118 (0.0035)*^b^* |
| SM (d43:1) | 0.0056 (0.0028) | -0.2876 (0.0620)*^c^* | 0.0071 (0.0038) |
| SM (d43:2) | 0.0094 (0.0028)*^b^* | -0.0571 (0.0625) | 0.0025 (0.0038) |
| SM (d43:2) A | 0.0080 (0.0028)*^a^* | -0.0389 (0.0614) | 0.0032 (0.0037) |
| SM (d43:2) B | 0.0050 (0.0029) | -0.0896 (0.0635) | 0.0051 (0.0039) |
| SM (d44:2) | 0.0186 (0.0027)*^c^* | -0.0606 (0.0597) | 0.0045 (0.0036) |
| TG (14:0/14:0/14:0) | -0.0011 (0.0029) | 0.0444 (0.0642) | 0.0076 (0.0039) |
| TG (40:0) | -0.0009 (0.0029) | 0.0315 (0.0644) | 0.0061 (0.0039) |
| TG (40:1) | 0.0010 (0.0029) | 0.0322 (0.0637) | 0.0065 (0.0039) |
| TG (42:0) | -0.0022 (0.0029) | 0.0221 (0.0643) | 0.0084 (0.0039) |
| TG (42:1) | -0.0011 (0.0029) | 0.0305 (0.0642) | 0.0083 (0.0039) |
| TG (42:2) | -0.0014 (0.0029) | 0.0142 (0.0642) | 0.0074 (0.0039) |
| TG (42:3) | 0.0025 (0.0028) | 0.0247 (0.0634) | 0.0063 (0.0039) |
| TG (44:0) | -0.0012 (0.0029) | 0.0513 (0.0637) | 0.0102 (0.0039)*^a^* |
| TG (44:1) | -0.0011 (0.0029) | 0.0583 (0.0637) | 0.0105 (0.0039)*^a^* |
| TG (44:2) | -0.0012 (0.0029) | 0.0135 (0.0640) | 0.0093 (0.0039) |
| TG (46:0) | -0.0008 (0.0028) | 0.0249 (0.0623) | 0.0107 (0.0038)*^a^* |
| TG (46:1) | -0.0009 (0.0028) | 0.0589 (0.0629) | 0.0121 (0.0038)*^a^* |
| TG (46:2) | -0.0008 (0.0029) | 0.0436 (0.0636) | 0.0108 (0.0039)*^a^* |
| TG (46:3) | -0.0009 (0.0029) | 0.0252 (0.0636) | 0.0109 (0.0039)*^a^* |
| TG (46:4) A | -0.0024 (0.0029) | -0.0542 (0.0636) | 0.0098 (0.0039)*^a^* |
| TG (48:0) | -0.0007 (0.0028) | 0.0020 (0.0615) | 0.0082 (0.0037) |
| TG (48:1) | 0.0011 (0.0027) | -0.0166 (0.0610) | 0.0113 (0.0037)*^a^* |
| TG (48:2) | -0.0008 (0.0028) | 0.0328 (0.0618) | 0.0131 (0.0038)*^b^* |
| TG (48:3) | -0.0006 (0.0028) | 0.0282 (0.0632) | 0.0117 (0.0039)*^a^* |
| TG (48:4) | -0.0003 (0.0029) | -0.0063 (0.0640) | 0.0084 (0.0039) |
| TG (48:5) | 0.0020 (0.0029) | -0.0001 (0.0641) | 0.0057 (0.0039) |
| TG (49:0) | -0.0014 (0.0028) | -0.1399 (0.0616)*^a^* | 0.0114 (0.0038)*^a^* |
| TG (49:1) | 0.0005 (0.0028) | -0.1189 (0.0618) | 0.0107 (0.0038)*^a^* |
| TG (49:2) | 0.0000 (0.0028) | -0.0424 (0.0613) | 0.0125 (0.0037)*^a^* |
| TG (49:3) | -0.0002 (0.0028) | -0.0196 (0.0621) | 0.0125 (0.0038)*^a^* |
| TG (50:0) | -0.0006 (0.0028) | -0.1957 (0.0621)*^b^* | 0.0098 (0.0038)*^a^* |
| TG (50:1) | 0.0025 (0.0027) | -0.1074 (0.0600) | 0.0086 (0.0037) |
| TG (50:2) | 0.0031 (0.0027) | -0.0488 (0.0595) | 0.0108 (0.0036)*^a^* |
| TG (50:3) | 0.0019 (0.0027) | -0.0313 (0.0610) | 0.0111 (0.0037)*^a^* |
| TG (50:4) | 0.0008 (0.0028) | -0.0804 (0.0630) | 0.0088 (0.0038) |
| TG (50:5) | 0.0016 (0.0028) | -0.0450 (0.0635) | 0.0081 (0.0039) |
| TG (50:6) | 0.0019 (0.0029) | 0.0112 (0.0636) | 0.0091 (0.0039) |
| TG (51:1) | 0.0005 (0.0028) | -0.1969 (0.0617)*^b^* | 0.0106 (0.0038)*^a^* |
| TG (51:2) | 0.0017 (0.0028) | -0.2087 (0.0613)*^b^* | 0.0102 (0.0037)*^a^* |
| TG (51:3) | 0.0019 (0.0028) | -0.2058 (0.0618)*^b^* | 0.0093 (0.0038) |
| TG (51:4) | 0.0027 (0.0028) | -0.1960 (0.0628)*^b^* | 0.0060 (0.0038) |
| TG (51:5) | 0.0028 (0.0028) | -0.1506 (0.0635)*^a^* | 0.0069 (0.0039) |
| TG (52:0) | -0.0045 (0.0028) | -0.2814 (0.0625)*^c^* | 0.0106 (0.0038)*^a^* |
| TG (52:1) | 0.0010 (0.0027) | -0.2239 (0.0610)*^c^* | 0.0104 (0.0037)*^a^* |
| TG (52:2) | 0.0068 (0.0027)*^a^* | -0.2242 (0.0596)*^c^* | 0.0070 (0.0036) |
| TG (52:3) | 0.0086 (0.0027)*^b^* | -0.2644 (0.0595)*^c^* | 0.0052 (0.0036) |
| TG (52:4) | 0.0063 (0.0028) | -0.2192 (0.0616)*^c^* | 0.0038 (0.0038) |
| TG (52:5) | 0.0051 (0.0028) | -0.1486 (0.0630)*^a^* | 0.0038 (0.0038) |
| TG (52:6) | 0.0037 (0.0028) | -0.0806 (0.0631) | 0.0080 (0.0038) |
| TG (53:1) | 0.0000 (0.0027) | -0.3210 (0.0609)*^c^* | 0.0096 (0.0037)*^a^* |
| TG (53:2) | 0.0022 (0.0027) | -0.3212 (0.0612)*^c^* | 0.0098 (0.0037)*^a^* |
| TG (53:3) | 0.0025 (0.0028) | -0.3059 (0.0619)*^c^* | 0.0078 (0.0038) |
| TG (53:4) | 0.0031 (0.0028) | -0.2774 (0.0625)*^c^* | 0.0062 (0.0038) |
| TG (53:5) | 0.0032 (0.0028) | -0.2089 (0.0631)*^b^* | 0.0043 (0.0038) |
| TG (54:0) | -0.0073 (0.0028)*^a^* | -0.3215 (0.0624)*^c^* | 0.0097 (0.0038)*^a^* |
| TG (54:1) | -0.0006 (0.0028) | -0.2987 (0.0622)*^c^* | 0.0081 (0.0038) |
| TG (54:2) | 0.0026 (0.0028) | -0.2953 (0.0614)*^c^* | 0.0077 (0.0037) |
| TG (54:3) | 0.0079 (0.0028)*^a^* | -0.3708 (0.0613)*^c^* | 0.0049 (0.0037) |
| TG (54:4) | 0.0067 (0.0028)*^a^* | -0.3567 (0.0619)*^c^* | 0.0044 (0.0038) |
| TG (54:5) A | 0.0046 (0.0028) | -0.2931 (0.0632)*^c^* | 0.0018 (0.0039) |
| TG (54:5) B | 0.0040 (0.0027) | -0.1564 (0.0600)*^a^* | 0.0126 (0.0037)*^b^* |
| TG (54:6) A | 0.0042 (0.0029) | -0.2085 (0.0640)*^b^* | -0.0012 (0.0039) |
| TG (54:6) B | 0.0023 (0.0027) | -0.1303 (0.0612) | 0.0114 (0.0037)*^a^* |
| TG (54:7) A | 0.0045 (0.0029) | -0.1765 (0.0641)*^a^* | -0.0040 (0.0039) |
| TG (54:7) B | 0.0030 (0.0028) | -0.1023 (0.0633) | 0.0069 (0.0039) |
| TG (54:8) A | 0.0042 (0.0028) | 0.0154 (0.0621) | 0.0047 (0.0038) |
| TG (54:8) B | 0.0037 (0.0028) | -0.0049 (0.0619) | 0.0103 (0.0038)*^a^* |
| TG (55:1) | 0.0014 (0.0028) | -0.2921 (0.0619)*^c^* | 0.0072 (0.0038) |
| TG (55:2) | 0.0032 (0.0028) | -0.3219 (0.0614)*^c^* | 0.0075 (0.0037) |
| TG (55:3) | 0.0040 (0.0028) | -0.3468 (0.0623)*^c^* | 0.0046 (0.0038) |
| TG (56:1) | -0.0002 (0.0028) | -0.2895 (0.0625)*^c^* | 0.0071 (0.0038) |
| TG (56:2) | 0.0025 (0.0028) | -0.3167 (0.0627)*^c^* | 0.0050 (0.0038) |
| TG (56:3) | 0.0040 (0.0028) | -0.3133 (0.0623)*^c^* | 0.0041 (0.0038) |
| TG (56:4) | 0.0054 (0.0028) | -0.2546 (0.0627)*^c^* | 0.0043 (0.0038) |
| TG (56:5) A | 0.0039 (0.0027) | -0.2189 (0.0612)*^c^* | 0.0098 (0.0037)*^a^* |
| TG (56:5) B | 0.0046 (0.0027) | -0.1895 (0.0595)*^b^* | 0.0145 (0.0036)*^b^* |
| TG (56:6) | 0.0046 (0.0027) | -0.2993 (0.0601)*^c^* | 0.0138 (0.0037)*^b^* |
| TG (56:7) A | 0.0022 (0.0028) | -0.2334 (0.0614)*^c^* | 0.0119 (0.0037)*^a^* |
| TG (56:7) B | 0.0079 (0.0027)*^a^* | -0.0414 (0.0605) | 0.0101 (0.0037)*^a^* |
| TG (56:8) A | 0.0033 (0.0028) | -0.0270 (0.0633) | 0.0084 (0.0039) |
| TG (56:8) B | 0.0074 (0.0028)*^a^* | -0.0845 (0.0623) | 0.0074 (0.0038) |
| TG (56:9) | 0.0051 (0.0028) | -0.0077 (0.0626) | 0.0096 (0.0038) |
| TG (57:1) | 0.0004 (0.0027) | -0.1373 (0.0612)*^a^* | 0.0056 (0.0037) |
| TG (57:2) | 0.0039 (0.0028) | -0.2886 (0.0623)*^c^* | 0.0050 (0.0038) |
| TG (58:1) | 0.0017 (0.0028) | -0.2659 (0.0630)*^c^* | 0.0048 (0.0038) |
| TG (58:10) | 0.0064 (0.0028) | 0.0335 (0.0617) | 0.0082 (0.0038) |
| TG (58:2) | 0.0024 (0.0028) | -0.3009 (0.0629)*^c^* | 0.0054 (0.0038) |
| TG (58:3) | 0.0031 (0.0028) | -0.2977 (0.0634)*^c^* | 0.0021 (0.0039) |
| TG (58:4) A | 0.0035 (0.0028) | -0.2887 (0.0632)*^c^* | 0.0021 (0.0039) |
| TG (58:5) | 0.0000 (0.0028) | -0.0975 (0.0630) | 0.0108 (0.0038)*^a^* |
| TG (58:6) | 0.0000 (0.0028) | -0.1426 (0.0619)*^a^* | 0.0115 (0.0038)*^a^* |
| TG (58:8) | 0.0086 (0.0028)*^b^* | -0.0243 (0.0618) | 0.0089 (0.0038) |
| TG (58:9) | 0.0064 (0.0028) | -0.0256 (0.0625) | 0.0096 (0.0038)*^a^* |
| TG (59:2) | 0.0028 (0.0028) | -0.2750 (0.0630)*^c^* | 0.0055 (0.0038) |
| TG (59:3) | 0.0045 (0.0028) | -0.2515 (0.0631)*^c^* | 0.0029 (0.0038) |
| TG (60:11) | 0.0051 (0.0028) | 0.0820 (0.0631) | 0.0091 (0.0038) |
| TG (60:2) | 0.0061 (0.0028) | -0.2697 (0.0618)*^c^* | 0.0023 (0.0038) |
| TG (60:3) | 0.0046 (0.0028) | -0.3002 (0.0631)*^c^* | 0.0033 (0.0038) |
| TG (60:4) | 0.0039 (0.0029) | -0.2695 (0.0635)*^c^* | 0.0012 (0.0039) |
| TG (60:6) | -0.0006 (0.0029) | 0.0260 (0.0636) | 0.0049 (0.0039) |
| TG (62:3) | 0.0076 (0.0028)*^a^* | -0.3066 (0.0630)*^c^* | 0.0000 (0.0038) |
| TG (62:4) | 0.0075 (0.0028)*^a^* | -0.3143 (0.0627)*^c^* | 0.0021 (0.0038) |
| TG (64:4) | 0.0052 (0.0028) | -0.1321 (0.0635) | 0.0008 (0.0039) |

**Legend:** Rows include regression coefficients (β) and standard errors from separate linear regression models with outcome of standardized lipid intensity. Each model includes age, sex, age*sex interaction, batch, and BMI as covariates. In all models, age is centered at the mean age of 48.3 years, and the reference category for sex is male. All *P*-values are adjusted for multiple testing using a Benjamini-Hochberg adjustment to control the false discovery rate among each set of 15 coefficients at 0.05. Age coefficients (β) can be interpreted as the expected change in standardized lipid intensity for a one-year increase in age, among men, after adjustment for batch and BMI. Sex coefficients (β) can be interpreted as the expected difference in standardized lipid intensity between women and men, at the mean age, after adjustment for batch and BMI (positive values indicate higher expected levels in women). Age*sex interaction coefficients (β) can be interpreted as the expected additional change in standardized lipid intensity for a one-year increase in age, among women (on top of the age coefficient for men), after adjustment for batch and BMI. Put more simply, add the age coefficient and age*sex interaction coefficient together to find the expected change in standardized lipid intensity for a one-year increase in age, among women, after adjustment for batch and BMI. Lipids (A) and (B) are resolved cis/trans stereoisomers of the same molecular lipid species.

Abbreviations: standard error (SE), triglycerides (TG), diacylglycerols (DG), phosphatidylcholines (PC), phospatidylethanolamines (PE), phosphatidylinositols (PI), phosphatidylglycerols (PG), lysophosphatidylethanolamines (LPE), lysophosphatidylcholines (LPC), sphingomyelins (SM), lactosylceramides (LCer), glucosylceramides (GlcCer), galactosylgalactosylceramides (GalGalCer), cholesterylesters (CE).

*^a^*adjusted *P*-value < 0.05

*^b^*adjusted *P*-value < 0.01

*^c^*adjusted *P*-value < 0.001
