# Supplementary material for: Age and sex are associated with the plasma lipidome: findings from the GOLDN study
Source: Lipids Health Dis. 2021 Apr 3;20:30. doi: 10.1186/s12944-021-01456-2 (PMC8019182; doi:10.1186/s12944-021-01456-2)
Supplement: Supplementary file 2 — Additional file 2. Correlation between total class intensities. [file 12944_2021_1456_MOESM2_ESM.docx]

**Additional File 2.** Correlation Between Total Class Intensities

**
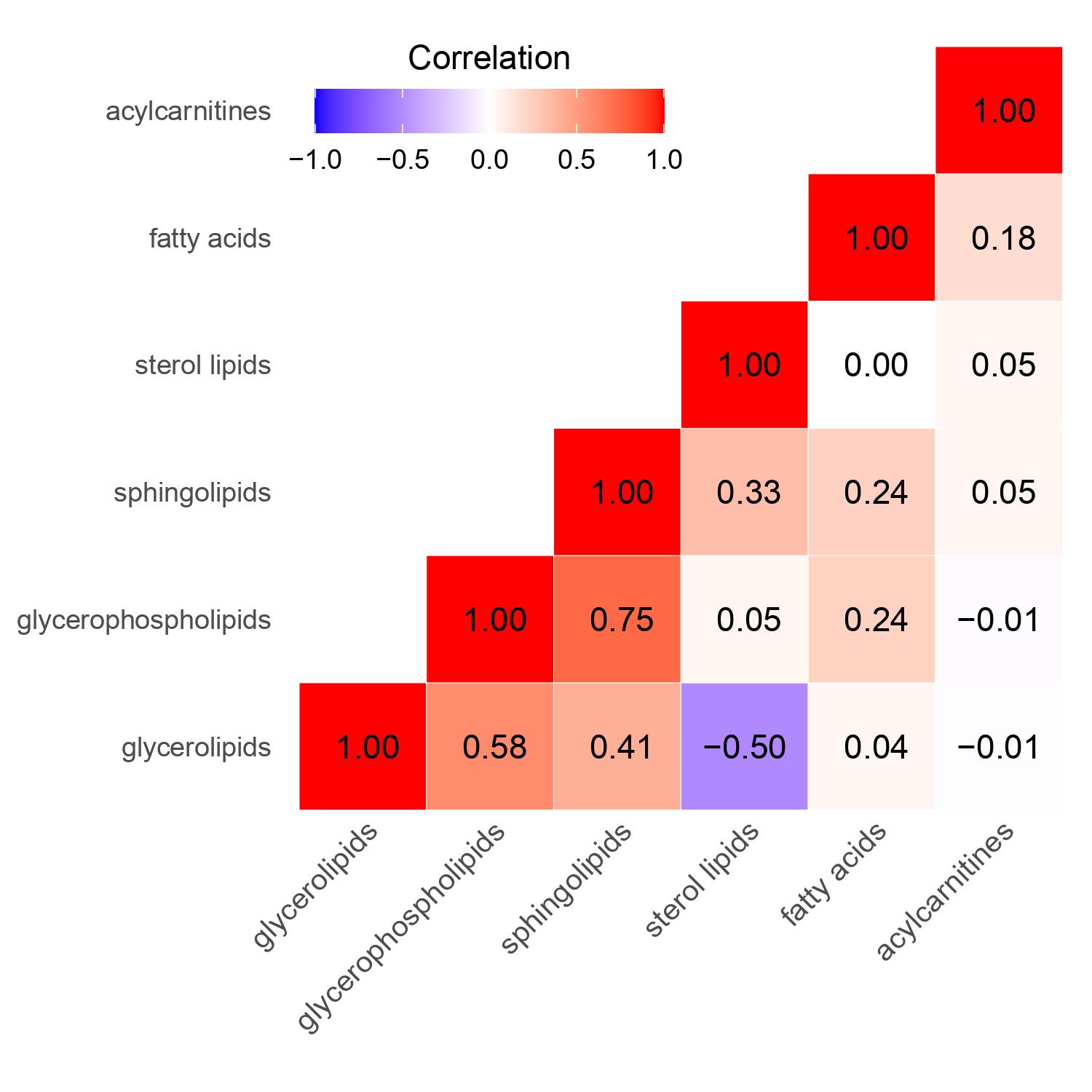
**

**Caption:** Heatmap shows the Pearson correlation between each pairwise combination of the six lipid classes. Darker colors represent stronger correlation, ranging from dark blue (-1.0) to dark red (1.0).
