# Supplementary material for: Age and sex are associated with the plasma lipidome: findings from the GOLDN study
Source: Lipids Health Dis. 2021 Apr 3;20:30. doi: 10.1186/s12944-021-01456-2 (PMC8019182; doi:10.1186/s12944-021-01456-2)
Supplement: Supplementary file 3 — Additional file 3. Diagnostic plots for regression models of lipid classes and subclasses. [file 12944_2021_1456_MOESM3_ESM.docx]

**Additional File 3.** Diagnostic Plots for Regression Models of Lipid Classes and Subclasses.

Class: glycerolipids


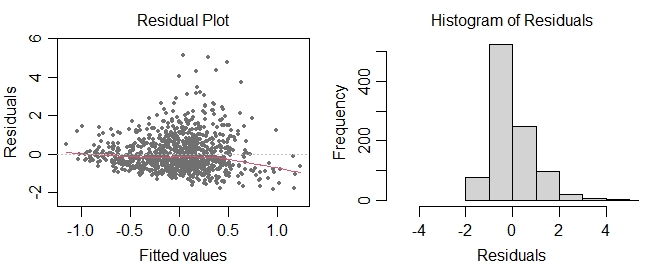


Class: glycerophospholipids


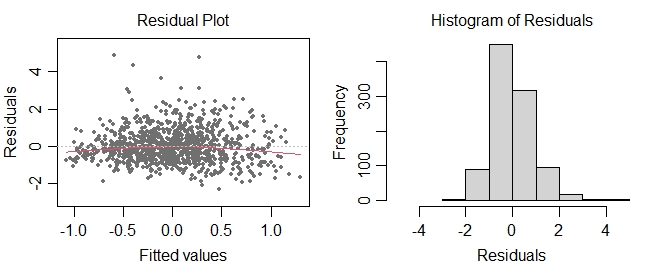


Class: sphingolipids


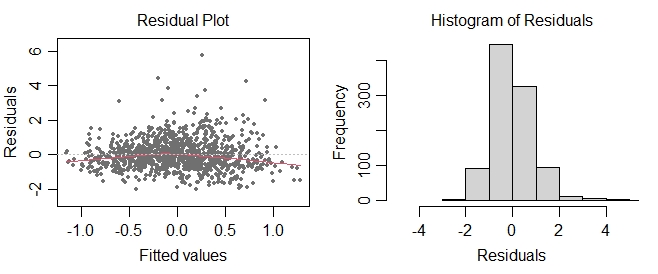


Class: sterol lipids


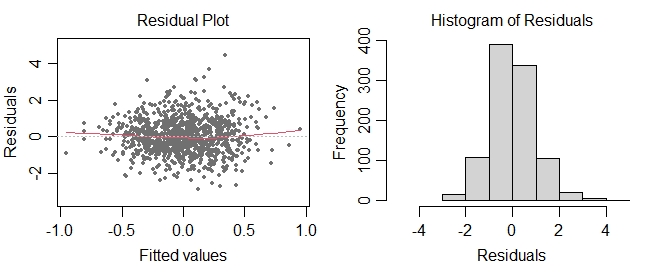


Class: fatty acids


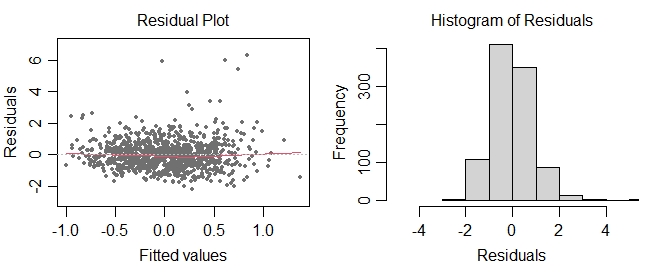


Class: acylcarnitines


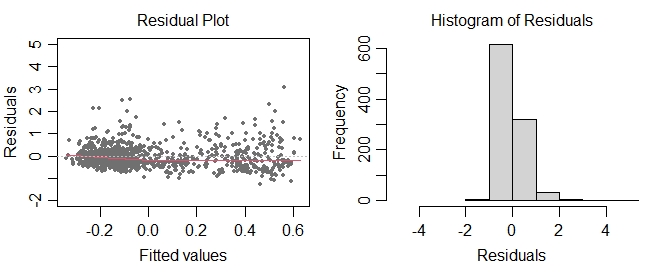


Subclass: triglycerides (TG)


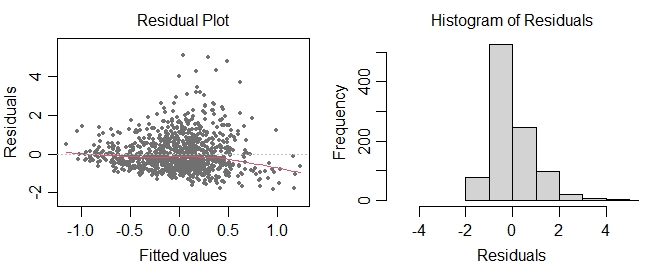


Subclass: diacylglycerols (DG)


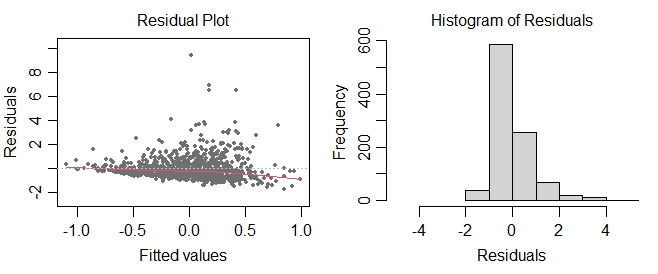


Subclass: phosphatidylcholines (PC)


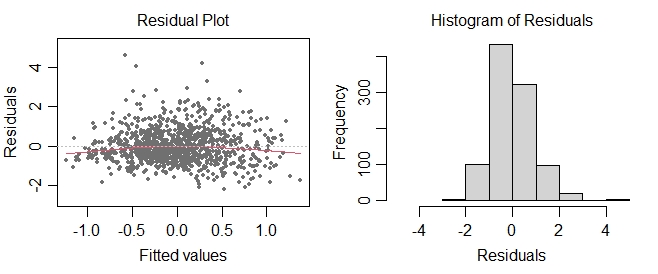


Subclass: phospatidylethanolamines (PE)


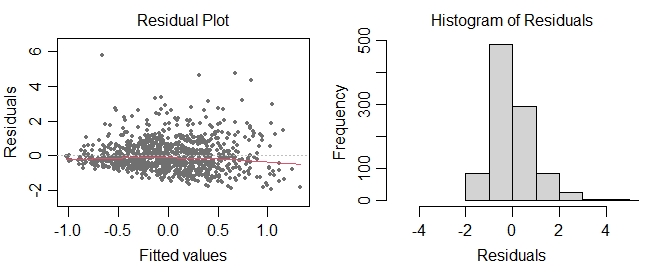


Subclass: phosphatidylinositols (PI)


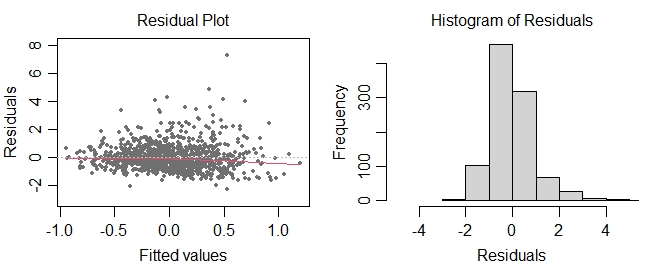


Subclass: phosphatidylglycerols (PG)


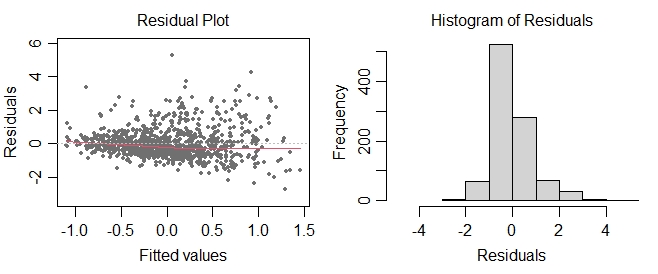


Subclass: lysophosphatidylethanolamines (LPE)


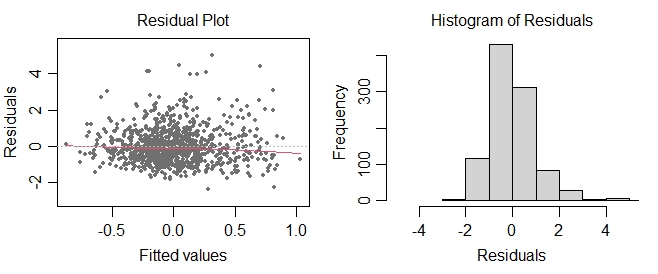


Subclass: lysophosphatidylcholines (LPC)


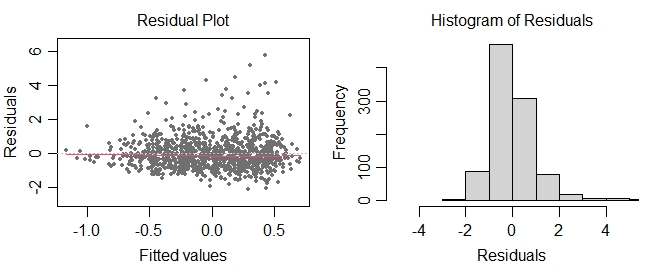


Subclass: sphingomyelins (SM)


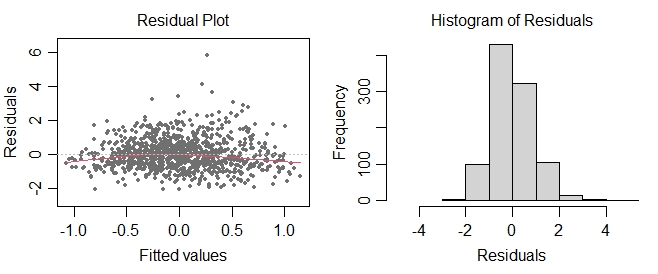


Subclass: ceramides


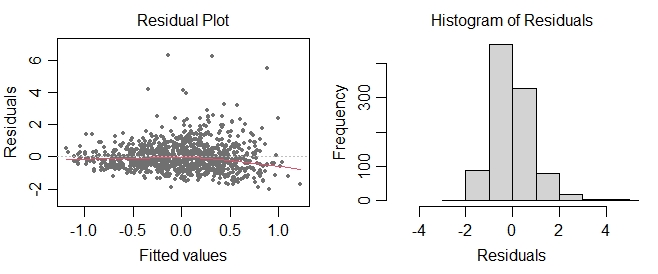


Subclass: lactosylceramides (LCer)


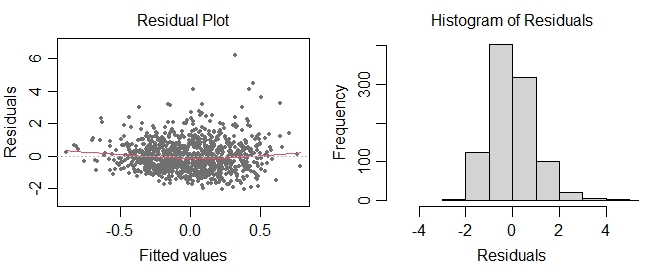


Subclass: glucosylceramides (GlcCer)


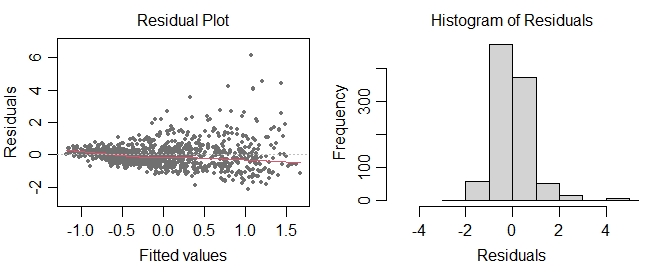


Subclass: galactosylgalactosylceramides (GalGalCer)


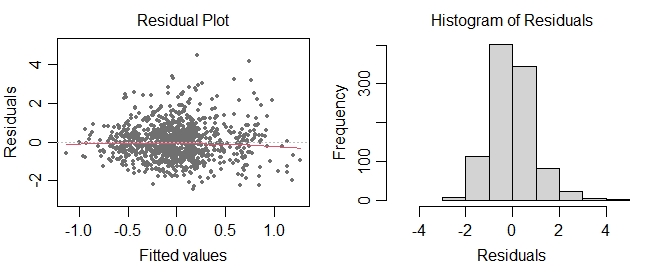


Subclass: cholesterol


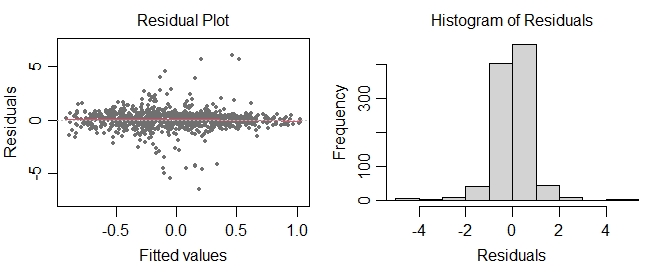


Subclass: cholesterylesters (CE)


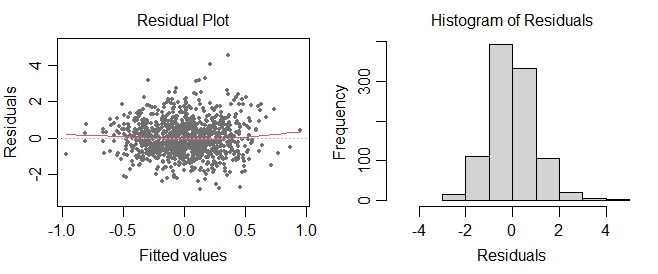


**Caption:** Each pair of diagnostic plots (left: residual plot, right: histogram of residuals) comes from the full linear regression model fit for each lipid class (model results in Table 2) and lipid subclass (model results in Table 3).
